# Vitamin A retinoic acid contributes to muscle stem cell and mitochondrial function loss in old age

**DOI:** 10.1172/jci.insight.183706

**Published:** 2025-03-25

**Authors:** Paula M. Fraczek, Pamela Duran, Benjamin A. Yang, Valeria Ferre, Leanne Alawieh, Jesus A. Castor-Macias, Vivian T. Wong, Steve D. Guzman, Celeste Piotto, Klimentini Itsani, Jacqueline A. Larouche, Carlos A. Aguilar

**Affiliations:** 1 Department of Biomedical Engineering,; 2 Biointerfaces Institute, and; 3 Program in Cellular and Molecular Biology, University of Michigan, Ann Arbor, Michigan, USA.

**Keywords:** Aging, Muscle biology, Stem cells, Adult stem cells, Muscle

## Abstract

Adult stem cells decline in number and function in old age, and identifying factors that can delay or revert age-associated adult stem cell dysfunction are vital for maintaining a healthy lifespan. Here we show that vitamin A, a micronutrient that is derived from diet and metabolized into retinoic acid, acts as an antioxidant and transcriptional regulator in muscle stem cells. We first show that obstruction of dietary vitamin A in young animals drives mitochondrial and cell cycle dysfunction in muscle stem cells that mimics old age. Next, we pharmacologically targeted retinoic acid signaling in myoblasts and aged muscle stem cells ex vivo and in vivo and observed reductions in oxidative damage, enhanced mitochondrial function, and improved maintenance of quiescence through fatty acid oxidation. We next detected that the receptor for vitamin A–derived retinol, stimulated by retinoic acid 6 or Stra6, was diminished with muscle stem cell activation and in old age. To understand the relevance of Stra6 loss, we knocked down Stra6 and observed an accumulation of mitochondrial reactive oxygen species, as well as changes in mitochondrial morphology and respiration. These results demonstrate that vitamin A regulates mitochondria and metabolism in muscle stem cells and highlight a unique mechanism connecting stem cell function with vitamin intake.

## Abstract 2

Our results shows that vitamin A-derived retinoic acid regulates muscle stem cell quiescence, mitochondrial function, and neutralizes oxidative damage that accumulates in old age.

## Graphical abstract

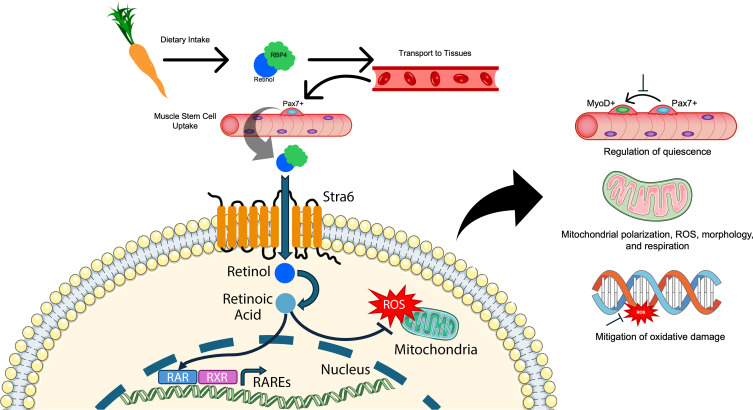

## Introduction

Skeletal muscle contains a small population of stem cells called muscle stem cells (MuSCs) or Pax7-expressing satellite cells (1). MuSCs resist entry into the cell cycle via a series of mechanisms driven by β-oxidation of fatty acids and oxidative phosphorylation (OXPHOS) resulting in low levels of reactive oxygen species (ROS) (2, 3). Impairments in MuSC ability to maintain quiescence have been demonstrated in old age, whereby a loss of OXPHOS, imbalances between mitochondrial fusion and fission, and dysregulation of mitophagy (4) result in oxidative damage and stem cell decline in number and function (5). MuSC dysfunction in old age has been shown to contribute to reductions in regeneration leading to persistent tissue damage, and structural and functional deficits (6–12). Therefore, it is critical to understand factors that promote MuSC metabolic and mitochondrial health to prevent pathological muscle remodeling in age (3).

A potential source of deleterious behavior that occurs in old-aged MuSCs (>22 months) is lower vitamin consumption (13). Vitamin A (VA), or retinol, is a diet-derived antioxidant that is metabolized into all-trans retinoic acid (RA) (14) and interacts with RA receptor–retinoid x receptor (RXR) transcription factors that bind at RA response elements. The interactive output of RA+RAR/RXRs results in transcription of genes related to development, vision, immunity, and metabolism (15, 16), and variations in RA levels have been shown to drive alterations in spatial patterning in development as well as changes in stem cell state and differentiation (17, 18). RA has also been shown to promote quiescence in hematopoietic stem cells, and to restrain human skeletal muscle progenitors from differentiation (19–22). The lack of VA and RA has also been associated with neuromuscular dysfunction (23, 24) and contributes to oxidative stress and mitochondrial dysfunction in T cells (25). However, there is little information connecting MuSC functionality with VA metabolism and in old age.

The membrane receptor STRA6 is the cellular gateway for VA entry and has been implicated in a variety of cellular functions including regulation of p53, Wnt, and mitochondrial pathways (26). STRA6 loss hinders VA homeostasis and retinoid-dependent processes (27), but there is no information on how STRA6 affects skeletal muscle, MuSCs, or changes in old age. Given the important links between mitochondria, ROS, and MuSCs, STRA6 may be critical for regulating RA and resisting oxidative stress in age.

Herein, we investigate the role of VA and RA signaling in MuSCs through a series of in vitro and in vivo experiments. We administered a VA-free diet to young animals and observed premature MuSC activation, oxidative DNA damage, and mitochondrial dysfunction akin to aging. Next, we pharmacologically upregulated RA signaling in myogenic progenitors and old-aged MuSCs ex vivo and in vivo and observed that we could enhance mitochondrial function and reduce oxidative stress. Last, we demonstrate that the VA receptor Stra6 is attenuated with activation and in old age and that *Stra6* loss perturbed mitochondrial dynamics resulting in accumulation of ROS and oxidative stress. These findings provide insights into the mechanisms driving MuSC mitochondrial health in old age with dietary-derived vitamins.

## Results

### In vivo depletion of VA drives premature activation of MuSCs.

**Figure 1:**
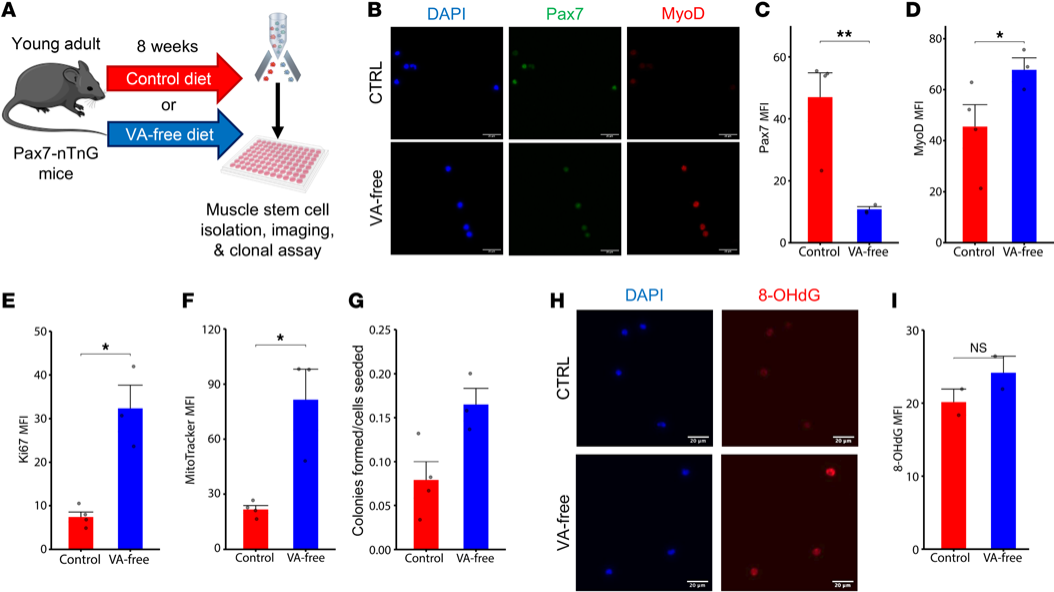
Dietary depletion of vitamin A induces premature activation and oxidative damage in muscle stem cells. (**A**) Experiment schematic whereby young mice were fed either a vitamin A–deficient or control diet for a total of 8 weeks, after which muscle stem cells were isolated and profiled. (**B**) Representative images of Pax7-nGFP and MyoD fluorescence of freshly isolated and fixed MuSCs from CTRL diet (top row) and VA-deficient diet (bottom row). DAPI, blue; Pax7, green; MyoD, red. Scale bar: 20 μm. (**C** and **D**) Quantification of mean fluorescence intensity of Pax7-nGFP and MyoD, respectively. Comparisons made via *t* test with *n* = 3–4 wells per diet. (**E** and **F**) Quantification of Ki67 and MitoTracker Deep Red, respectively, in MuSCs fixed immediately after isolation from mice receiving CTRL diet (red) or VA-free diet (blue). Comparisons made via *t* test with *n* = 3–4 wells per diet. (**G**) Proportion of colony-forming single MuSCs isolated from mice receiving CTRL diet (red) or VA-free diet (blue) after 5 days in growth conditions. Comparisons made via *t* test with *n* = 4 mice (60 wells per mouse) for the control diet, and *n* = 3 mice (95 wells per mouse) for VA-free diet. (**H**) Representative images of 8-OHdG fluorescence of freshly isolated and fixed MuSCs from CTRL diet (top row) and VA-free diet (bottom row). DAPI, blue; 8-OHdG, red. Scale bar: 20 μm. (**I**) Quantification of mean fluorescence intensity of 8-OHdG in MuSCs fixed immediately after isolation from mice receiving CTRL diet (red) or VA-free diet (blue). *n* = 6 image fields (across 2 mice) per diet. Data are shown as mean ± SEM (*P < 0.05, **P < 0.01, ***P < 0.001, ****P < 0.0001 for all comparisons).

To determine the effect of the lack of VA and concomitant RA signaling in MuSCs in vivo, we administered a VA-free diet ad libitum for 8 weeks to a MuSC lineage tracing mouse model (Pax7^CreERT2^-Rosa26^nTnG^ [Pax7-nTnG]) (28). After 8 weeks, MuSCs were isolated from the quadriceps and gastrocnemius muscles via using fluorescence activated cell sorting (FACS; Supplemental Figure 1A; supplemental material available online with this article; https://doi.org/10.1172/jci.insight.183706DS1) and were profiled (Figure 1A). Since previous studies have shown that RA restrains MuSCs from differentiation in vitro, we first evaluated changes in MuSC activation (20, 29, 30). Freshly isolated VA-free MuSCs displayed reductions in Pax7 (*P* < 0.01), and increased MyoD compared with controls (*P* < 0.05; Figure 1, B–D). A higher overall proportion of VA-depleted MuSCs formed myogenic colonies compared with control MuSCs (*P* < 0.05; Figure 1G), suggesting an increased propensity to enter the cell cycle. In line with these observations, we detected increases in Ki67 (*P* < 0.05) as well as increased amounts of mitochondria (MitoTracker Deep Red FM; *P* < 0.05) in MuSCs isolated from VA-free diet (Figure 1, E and F). To further examine if VA depletion promoted accumulation of ROS and oxidative stress–related damage, we stained for 8-hydroxyguanosine (8-OHdG), a form of DNA lesion induced by exposure to ROS (31). VA-free MuSCs displayed increased levels of 8-OHdG when compared with controls (*P* < 0.05; Figure 1, H and I). These results confirm that VA depletion prompted MuSCs to shift away from quiescence and toward premature activation.

### Lack of VA disrupts MuSC metabolism.

**Figure 2:**
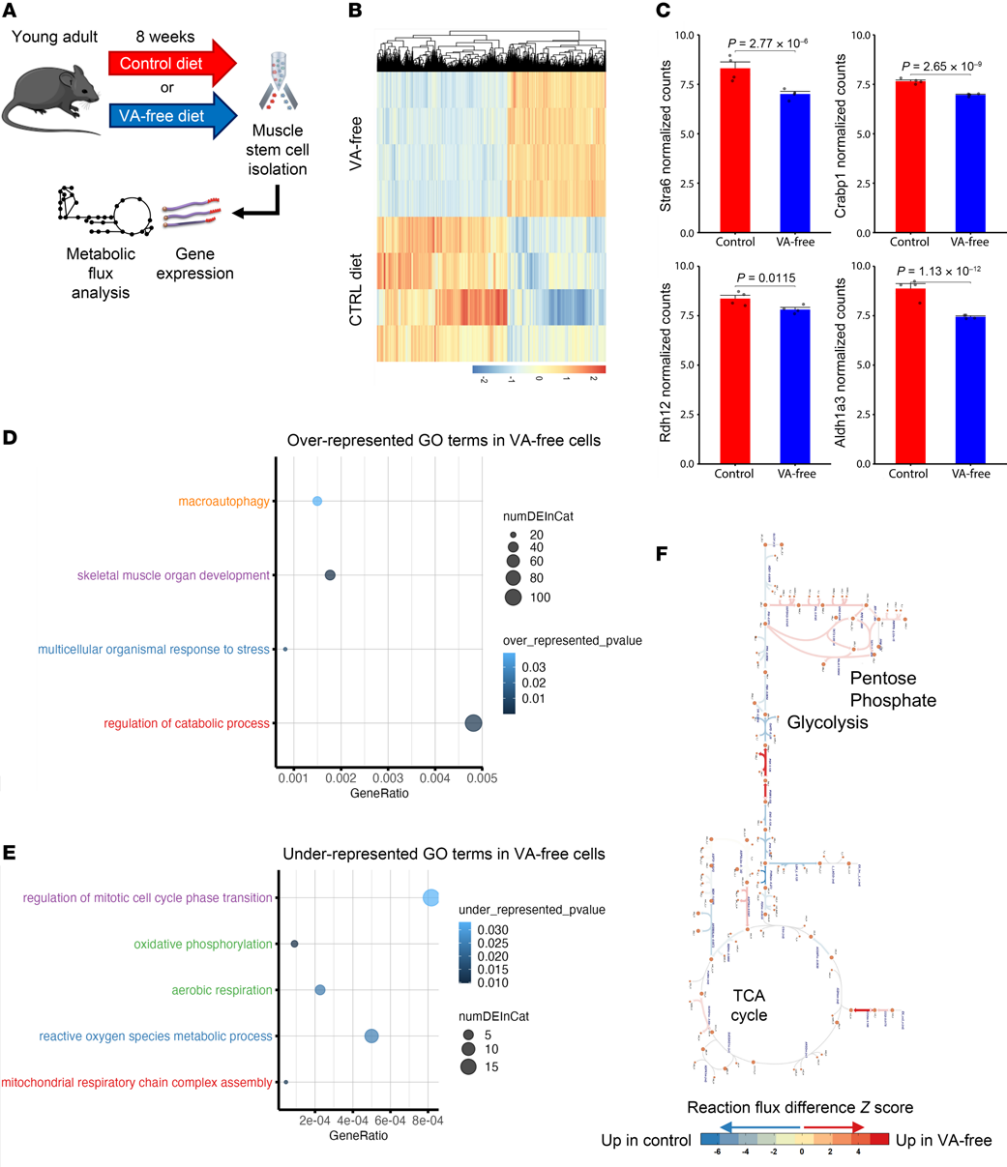
Vitamin A depletion disrupts muscle stem cell metabolism, mitochondria, and cell cycle regulation. (**A**) Experiment schematic: young mice were fed either a vitamin A–deficient or control diet for a total of 8 weeks, after which muscle stem cells were isolated via FACS and RNA was extracted for RNA-Seq, followed by differential gene expression, GO term enrichment, and metabolic flux model analyses. (**B**) Heatmap of *z* scores for all differentially expressed genes with *P*_adj_ < 0.05 from muscle stem cells isolated from control and vitamin A–free diet-fed young mice. (**C**) Bar plots of selected differentially expressed genes related to vitamin A metabolism and RA signaling. Data are shown as mean ± SEM. (**D**) Bubble plots of selected overrepresented GO terms across differentially expressed genes. (**E**) Bubble plot of selected underrepresented GO terms across differentially expressed genes. (**F**) Escher map of statistically significant metabolic fluxes predicted by metabolic flux model. Red, enriched fluxes in vitamin A–free diet; blue, enriched fluxes in control diet.

To gain further insights into changes in MuSCs from a VA-free diet, we performed RNA-Seq of FACS-isolated MuSCs from mice fed either a VA-free diet or control chow (Figure 2A). We observed excellent reproducibility between libraries (*r* ≥ 0.93) from different conditions (Supplemental Figure 1B), and principal component analysis revealed clustering of replicates and distinction between MuSCs from a control and VA-free diet (Supplemental Figure 1C). Differential expression analysis revealed 4,149 genes underwent a change due to VA-free diet (Figure 2B and Supplemental Table 1), and multiple components of the RA signaling pathway were attenuated such as dehydrogenases that oxidize retinol into RA (e.g., *Adh1*, *Aldh1a3*) and cellular RA binding proteins (e.g., *Crabp1*) that carry RA into the nucleus (Figure 2C). Additionally, the surface receptor that mediates cellular uptake of retinol (stimulated by RA gene 6 or *Stra6*) decreased in expression with VA-free diet (Figure 2C). We also detected several senescence-related genes in VA-free diet MuSCs including cytokines *Il-6*, *Il-15*, and *Ccl4* (Supplemental Table 2). GO term enrichment analysis of differentially expressed genes showed overrepresentation of autophagy, MuSC activation, and differentiation for VA-free diet MuSCs. In contrast, underrepresented terms in VA-free diet MuSCs included cell cycle and ROS regulation and multiple types of mitochondrial processes including mitochondrial membrane organization, OXPHOS, and mitochondrial ROS mitigation (Figure 2, D and E, and Supplemental Figure 1D).

To further probe how diet-induced changes in gene expression affected metabolic flux, we applied genome-scale metabolic modeling to our RNA-Seq data (32). The metabolic model predicted VA-free diet–increased flux through L-lactate dehydrogenases and retinol dehydrogenases that contribute to NAD^+^/NADH balance and nucleotide synthesis as well as sense changes in available lipids and steroids (Figure 2F and Supplemental Figure 1E). The model also predicted increased flux through the pentose phosphate pathway (Figure 2F), which is consistent with activation of nicotinamide adenine dinucleotide phosphate (NADPH) oxidase family and changes in redox signaling that promotes MuSC activation (33, 34). In contrast, MuSCs from control diet were enriched for pyruvate kinase, which has been shown to block ROS in myogenic progenitors (35). Together, these results suggest that depleting VA promotes changes in MuSC metabolism that reduce maintenance of quiescence and signaling through mitochondria to neutralize oxidative stress.

### Loss of dietary VA does not alter MuSC-mediated regeneration.

To investigate whether dietary depletion of VA reduced the regenerative potential of MuSCs, we administered a muscle injury via intramuscular injection with barium chloride (BaCl_2_). Dissection and IHC of tibialis anterior (TA) cross sections before injury revealed no statistically significant variations in cross-sectional myofiber area between control or VA-free diet (Supplemental Figure 2, A and B). We also did not detect a significant change in the number of monocytes and macrophages (CD68^+^) or neutrophils (Ly6G^+^), nor did we detect one in the total number of MuSCs for VA-free diet when compared with controls (Supplemental Figure 2, C–E). Seven days after injury, we detected no significant change in myofiber size and number of regenerating myofibers measured by centrally located nuclei (Supplemental Figure 2B). However, increases in both CD68^+^ and Ly6G^+^ cells were observed after injury for VA-free diet, which is consistent with the known role of VA as an antiinflammatory micronutrient (36).

To understand if MuSC changes in regenerative potential from VA-free diet occurs over a longer time scale, we assessed response to BaCl_2_ injury at 28 days after injury (Supplemental Figure 3A). In line with previous observations, no significant differences were identified in the cross-sectional area (CSA) of all myofibers (Supplemental Figure 3B) or in the number or percentage of myofibers with MuSC-derived engraftment between control and VA-free diet (Supplemental Figure 3, C and D) or centrally nucleated fibers (Supplemental Figure 3E). Neither did we detect differences in the cell density of CD68^+^ macrophages (Supplemental Figure 3F) nor MuSCs for VA-free diet when compared with controls (Supplemental Figure 3G). Overall, these results suggest that the lack of VA does not significantly affect acute response to regeneration from MuSCs.

### Targeting RA signaling alters mitochondrial function and morphology of MuSCs.

**Figure 3:**
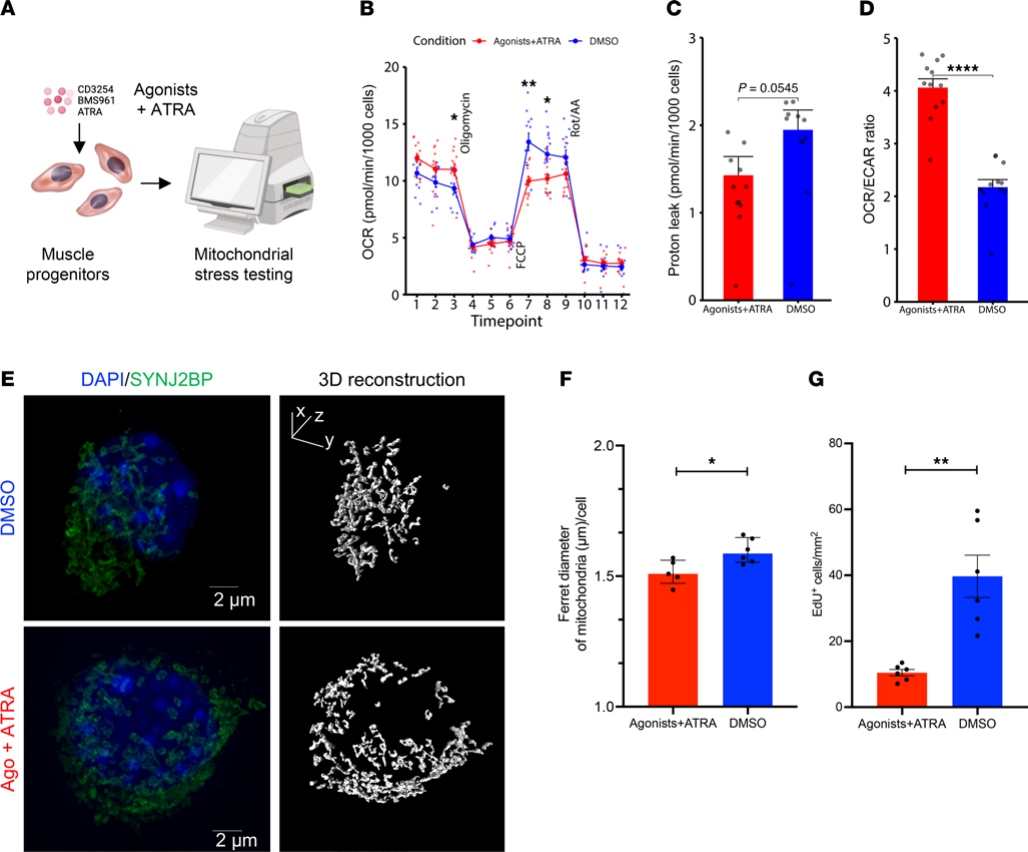
Small molecule agonists targeting retinoic acid signaling improves mitochondrial function and reduces reactive oxygen species. (**A**) Schematic depicting strategy to upregulate RA signaling by using Rarγ and Rxrα agonists (CD3254, BMS961) and ATRA as a ligand (each 100 nM). (**B**) Line graphs of oxygen consumption rate (OCR) measured via Seahorse XFe96 Mito Stress Test in C2C12s treated with ATRA and agonists (red, *n* = 12 wells) and DMSO vehicle control (blue, *n* = 12 wells) after injections of oligomycin, FCCP, and rotenone/antimycin A. (**C** and **D**) Quantification of proton leak and OCR/ECAR ratio, respectively, in C2C12s treated with ATRA and agonists (red) and DMSO vehicle control (blue). Comparisons of Seahorse Mito Stress parameters were made via *t* test. (**E**) A 3D projection and 3D reconstruction of single MuSCs from Pax7^CreERT2^-Rosa26^CAG–LSL–EGFP–3xHA–OMM^ mice showing individual mitochondria after cellular treatment with DMSO control (top) or ATRA and agonists (bottom). Scale bar: 2 μm. (**F**) Quantification of 3-dimensional Feret diameter between MuSCs treated with DMSO control and ATRA and agonists groups. Comparison made via Mann-Whitney *U* test for nonparametric distributed data with *n* = 4 wells per treatment. Data represented as median with interquartile range. (**G**) Quantification of cellular density of EdU^+^ MuSCs between MuSCs treated with DMSO vehicle control (blue) or agonists and ATRA (red). Comparison made via *t* test with *n* = 6 wells per treatment.

We have previously demonstrated that MuSCs lose RAR and RXR gene expression in old age (37), and dietary loss of this exogenous antioxidant in young animals impinged on MuSC mitochondria. To further understand how targeting RAR/RXRs promotes mitochondrial health in myogenic cells, we delivered a cocktail of small molecule agonists specific to RARγ (BMS961) and RXRα (CD3254), as well as the ligand (ATRA) to C2C12 myoblasts (Figure 3A). We previously observed that *Rarg* and *Rxra* are among the most significantly downregulated RA-responsive genes in aged MuSCs, when compared with young cells (37), and *Rarg* has been shown to be essential in the regulation of most genes involved in retinol metabolism and RA signaling, including *Stra6* (38). We administered the small molecule cocktail to C2C12s and detected increased expression of the RA-responsive genes *Rarg* and *Rarb* as well as *Stra6* for cells treated with the small molecule cocktail (Supplemental Figure 4, A–C). We also detected that the orphan receptor peroxisome proliferator activated receptor γ (*Pparg*) increased in expression, as well as the NADH:ubiquinone oxidoreductase subunit A2 (*Ndufa2*), a critical catalytic component of mitochondrial respiratory chain and OXPHOS system (Supplemental Figure 4, D and E). To further validate the delivery of the small molecule cocktail affected mitochondrial function, we performed a Seahorse XF Mito Stress Test assay (Figure 3B) and observed increases in basal respiration and ATP production as well as reductions in proton leak for treated C2C12 cells (Figure 3C and Supplemental Figure 4, F–I). Treatment also increased coupling efficiency (rate of ATP production/rate of basal respiration) and oxygen consumption rate/extracellular acidification rate (OCR/ECAR), indicating increased ATP production through OXPHOS (Figure 3D). Notably, we found that improvements in activation restraint, mitochondria, and oxidative damage appeared strongest when ATRA and CD3254/BMS961 were combined rather than when they were analyzed individually (Supplemental Figure 4, J–L). These results show that increasing RA signaling in myogenic cells can promote mitochondrial function through oxidative metabolism. To further understand how MuSC changes in mitochondria and maintenance of quiescence that are enacted with treatment, we utilized a MuSC reporter mouse that harbors an enhanced green fluorescent protein (EGFP) in the outer mitochondrial membrane (OMM) of Pax7-expressing cells (Pax7^CreERT2^-Rosa26^CAG–LSL–EGFP–3xHA–OMM^ [Pax7-MitoTAG]; Figure 3E) (39). We isolated MuSCs from young muscles (4 months) and treated cells with or without the small molecule cocktail as above. Immunostaining the cells and analysis by 3D volume reconstruction showed reductions in Feret diameter (Figure 3F), which is consistent with previous observations that activated MuSCs contain larger-sized mitochondria (4). We further confirmed treatment with the small molecule cocktail reduced activation in Pax7-MitoTAG MuSCs by measuring EdU incorporation, and we detected reductions in EdU uptake for treated cells (Figure 3G and Supplemental Figure 4M). Combined with the results above, these findings show that increases in RA signaling reduce activation through mitochondria. 

### Repletion of RA signaling reduces oxidative stress and promotes metabolism supportive of quiescence in MuSCs.

**Figure 4:**
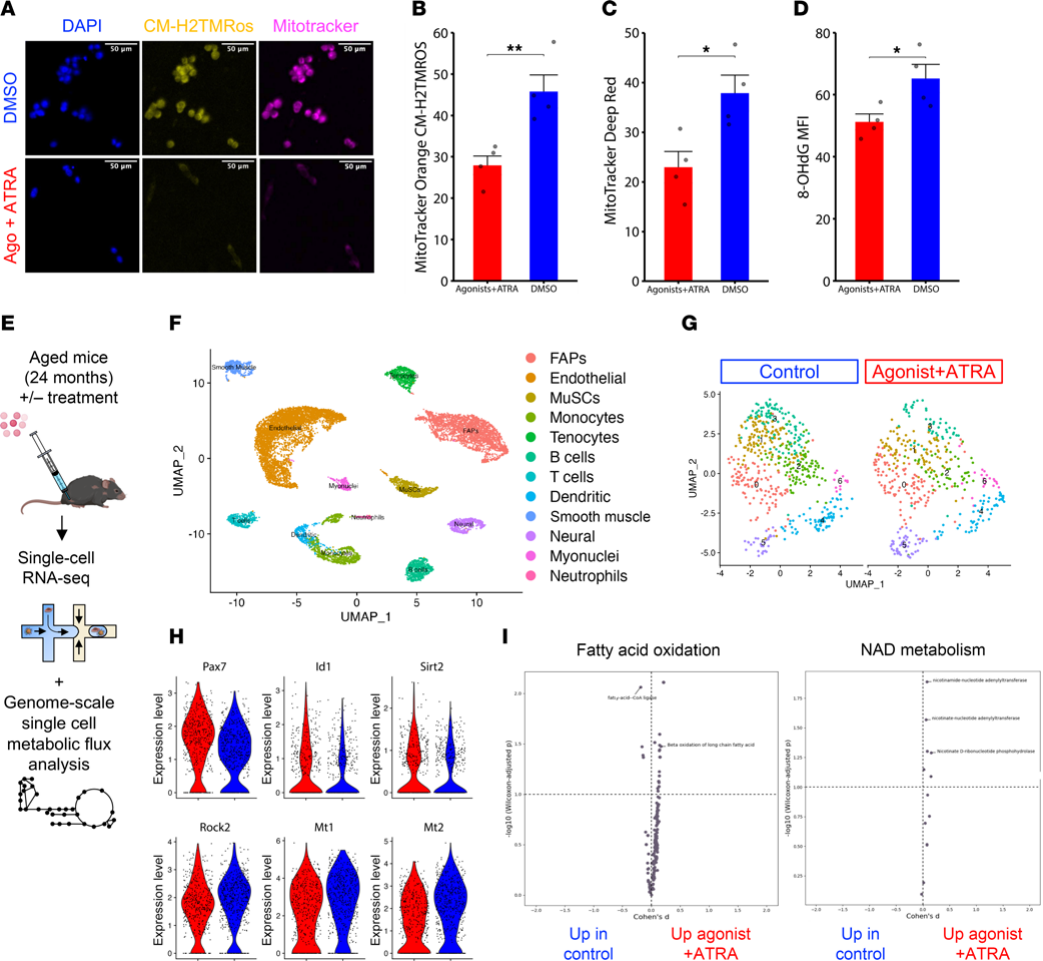
Repletion of retinoic acid signaling reduces oxidative stress and promotes metabolism supportive of quiescence in muscle stem cells. (**A**) Representative images of mitochondrial ROS labeled with MitoTracker Orange CM-H2TMRos (yellow) and total mitochondria labeled with MitoTracker Deep Red (magenta) in aged MuSCs treated with DMSO vehicle control (top) or 100 nM agonists and ATRA (bottom). DAPI counterstain is shown in blue. Scale bar: 50 μm. (**B**–**D**) Quantification of MitoTracker Orange CM-H2TMRos, MitoTracker Deep Red, and 8-OHdG mean fluorescence intensity between aged MuSCs treated with DMSO vehicle control or agonists and ATRA. Data are shown as mean ± SEM. (**E**) Schematic depicting strategy to upregulate RA signaling in aged mice through intramuscular injections of CD3254, BMS961, and ATRA, followed by scRNA-Seq of muscle cell suspensions and genome-scale metabolic flux balance analysis of single-cell transcriptomes. (**F**) Annotated UMAP of cell clusters obtained in the merged scRNA-Seq dataset of treated and untreated aged cells. (**G**) Subclustering of the MuSC subset in the treated (right) and untreated (left) samples. (**H**) Violin plots of selected differentially expressed genes found via DESeq2 (*P* < 0.1 and |log_2_FC| > 0.15). Top row: Pax7, Id1, Sirt2. Bottom row: Rock2, Mt1, Mt2. (**I**) Volcano plot of differentially expressed metabolic fluxes in the fatty acid oxidation and NAD metabolism subsystems determined by Compass analysis. Data are shown as mean ± SEM. Statistical comparisons were made via paired *t* test (*P < 0.05, **P < 0.01).

To determine if rescue of RA signaling in old-aged MuSCs improved cellular state and mitochondrial health, we isolated MuSCs from uninjured old-aged limb muscles (24 months) and treated old-aged MuSCs ex vivo with the same small molecule cocktail. We measured mitochondrial ROS levels by MitoTracker Orange CM-H_2_TMRos (40) and overall mitochondrial density (MitoTracker Deep Red FM). We detected reductions in mitochondrial ROS and total mitochondrial density for treated cells when compared with controls (Figure 4, A–C). To determine if treatment reduced oxidative DNA lesions, we stained for 8-OHdG and found reductions in treated MuSCs (Figure 4D). As above, the combined action of ATRA and CD3254/BMS961 rather than individual treatment resulted in the strongest increases in STRA6 and reductions in MyoD, mitochondrial ROS, and mitochondrial density (Supplemental Figure 4, N–Q). These results show that the pathological degeneration of MuSCs in old age, which is linked to dysfunctional mitochondria and oxidative damage, can be partially alleviated by targeting RA signaling (41).

To further determine how targeting RA signaling affected old-aged MuSCs in vivo, we intramuscularly injected the same small molecule cocktail every other day for 7 days to old-aged TA muscles (Figure 4E). We then performed single-cell RNA-Seq (scRNA-Seq) of mononucleated cells after therapy and generated 13,859 single cells after filtering (6,906 from aged treated and 6,953 from aged control cells) with an average of 2,113 genes and 5,841 unique molecular identifiers per cell (Supplemental Figure 5A). We reduced the dimensionality of the datasets using Uniform Manifold Approximation and Projection (UMAP) and performed Louvain clustering followed by annotation of cell types using marker genes (Figure 4F and Supplemental Figure 5B). This analysis revealed 12 cell types and similar recovery of cell types with previously published single-cell atlases from muscle (Supplemental Figure 5B) (28, 42). We detected consensus among cell types across samples (Supplemental Figure 5C) and increased fractions of neutrophils and tenocytes for controls compared with treated muscles (Supplemental Figure 5D). To glean insights into molecular responses of MuSCs with treatment, we reclustered MuSCs and found upregulated genes from treatment included *Pax7* and inhibitor of differentiation 1 and 3 (*Id1*, *Id3*), which are negative regulators of MyoD (43, 44) (Figure 4G and Supplemental Figure 5, E and F). We also detected increased expression of sirtuin 2 (*Sirt2*), a fatty-acid oxidation sensitive enzyme that regulates NAD^+^ availability, for treated MuSCs when compared with controls (Figure 4H) (45, 46). Conversely, upregulated genes in untreated cells included metallothioneins 1 and 2 (*Mt1*, *Mt2*), markers of oxidative stress, and *Rock2*, a negative regulator of mitophagy (47, 48). GO term enrichment analysis showed that treatment reduced apoptotic pathways, cell cycle entry, and TOR signaling (Supplemental Figure 5G). To gain deeper insights into alterations in metabolic flux from treatment, we utilized Compass, an algorithm to determine changes in metabolism from scRNA-Seq datasets (49). Consistent with our previous measurements, we detected increases in fatty acid oxidation and β-oxidation of long-chain fatty acids, as well as NAD metabolism in treated MuSCs when compared with untreated MuSCs (Figure 4I and Supplemental Figure 5H). Combining these results further suggests that targeting RA signaling augments metabolic health and reduces oxidative stress in MuSCs.

### MuSC intake of VA is mediated by Stra6, which decreases with activation and age.

**Figure 5:**
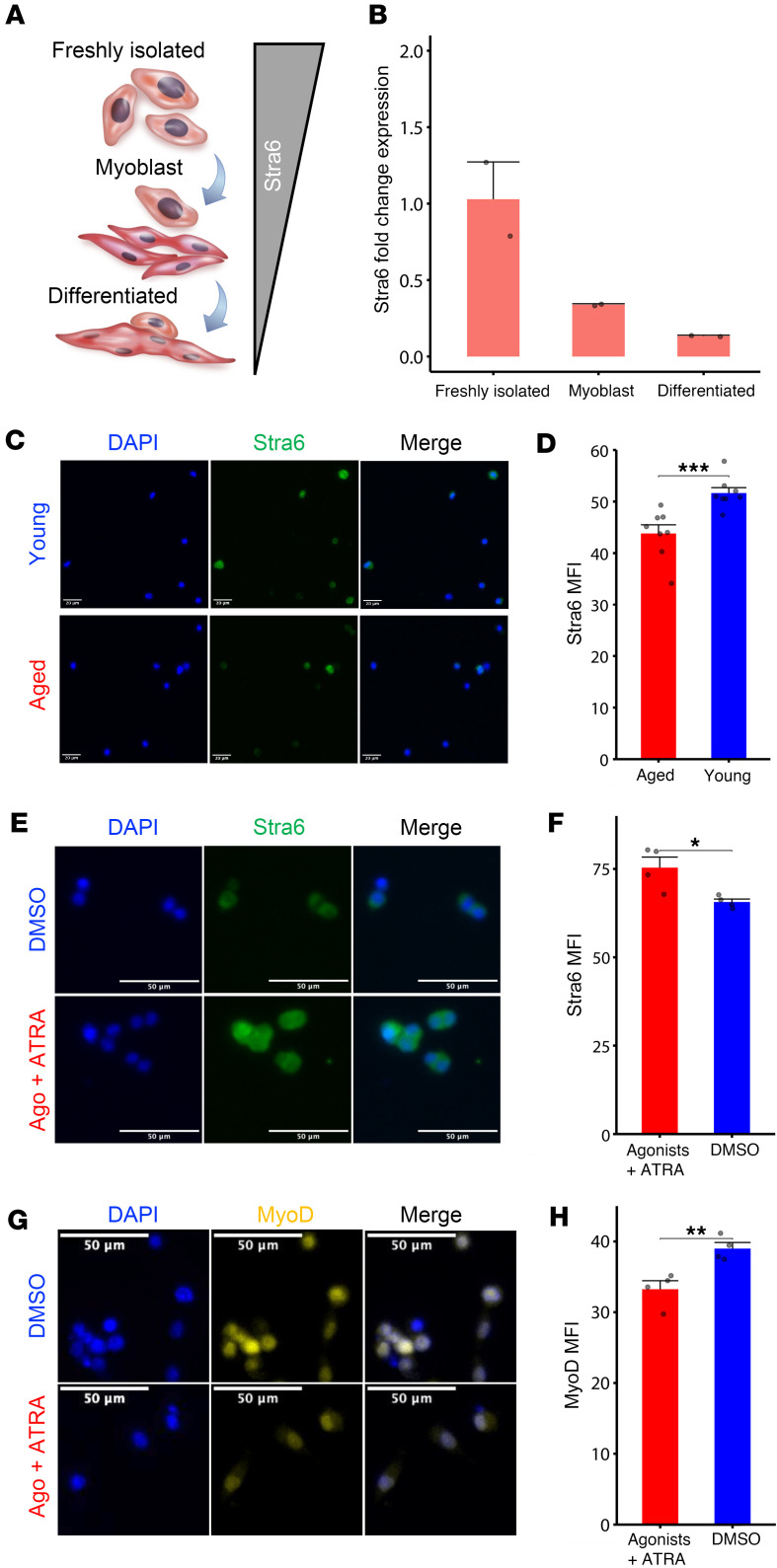
The vitamin A receptor Stra6 is attenuated with stem cell activation and aging. (**A**) Schematic depicting how Stra6 expression decreases with muscle stem cell activation and differentiation. (**B**) Quantification of Stra6 expression fold change (relative to Gapdh) in freshly isolated MuSCs, activated myoblasts (72 hours in culture with growth medium), and differentiated myotubes (an additional 72 hours in culture with differentiation medium). qPCR was run using 2 biological replicates and 2 technical replicates per time point. (**C**) Representative immunofluorescence images of Stra6 from muscle stem cells isolated from freshly isolated young (3–4 months) and aged mice (22 months). Tissues from 2 biological replicates (C57BL/6 females) were used per age group and pooled during MACS isolation before seeding into a 96-well plate, fixing, and labeling for Stra6. (**D**) Quantification of Stra6 mean fluorescence intensity comparing age groups using a t test. *n* = 8 wells were imaged per age group. (**E**) Representative images of Stra6 immunofluorescence staining in old-aged MuSCs (pooled from *n* = two 24-month-old female C57BL/6 mice) treated with DMSO vehicle control (top) or agonists and ATRA (bottom) for 3 days. Stra6, green; DAPI, blue. Scale bar: 50 μm. (**F**) Quantification of Stra6 mean fluorescence intensity between old-aged MuSCs treated with DMSO vehicle control (blue) or agonists and ATRA (red). Comparison made via *t* test with *n* = 4 wells per treatment. (**G**) Representative images of MyoD immunofluorescence staining in old-aged MuSCs treated with DMSO vehicle control (top) or agonists and ATRA (bottom). MyoD, yellow; DAPI, blue. Scale bar: 50 μm. (**H**) Quantification of MyoD mean fluorescence intensity between aged MuSCs treated with DMSO vehicle control or agonists and ATRA. Comparison made via *t* test with *n* = 4 wells per treatment.

The membrane receptor STRA6 is the cellular gateway for retinol entry, and our data show MuSCs lose *Stra6* gene expression after VA-free diet (Figure 2C). We first investigated whether *Stra6* expression changes using qPCR after 3 conditions: (a) freshly FACS-isolated MuSCs (quiescent) from uninjured limb muscles, (b) in vitro culture in activating conditions for 3 days (myoblast), and (c) 3 days after differentiation and fusion into myotubes (differentiated). In line with our previous observations, we detected that *Stra6* expression was highest for freshly isolated MuSCs, reduced in activated myoblasts, and lowest in differentiated myotubes (Figure 5, A and B). These results suggest that VA intake through STRA6 may participate in MuSC maintenance of quiescence. Given old-aged MuSCs have been demonstrated to lose quiescence and display premature activation, we quantified protein levels of STRA6 on freshly isolated young (3–4 months) and old-aged (22 months) MuSCs from young and aged mouse hind limb muscles. We found decreases in STRA6 levels in aged MuSCs, in accordance with our previous RNA findings where we previously observed a loss in *Stra6* gene expression from uninjured old-aged MuSCs (37) (Figure 5, C and D). Since STRA6 expression is enhanced by the binding of the RARγ/RXRα heterodimer to its promoter (50), we treated old-aged MuSCs as above, and confirmed increased Stra6 (Figure 5, E and F), which also coincided with reductions in MyoD (Figure 5, G and H). These results suggest that an attenuation of STRA6-mediated VA transport and RA signaling occurs during MuSC activation.

### Stra6 loss alters mitochondrial state, morphology, and function.

**Figure 6:**
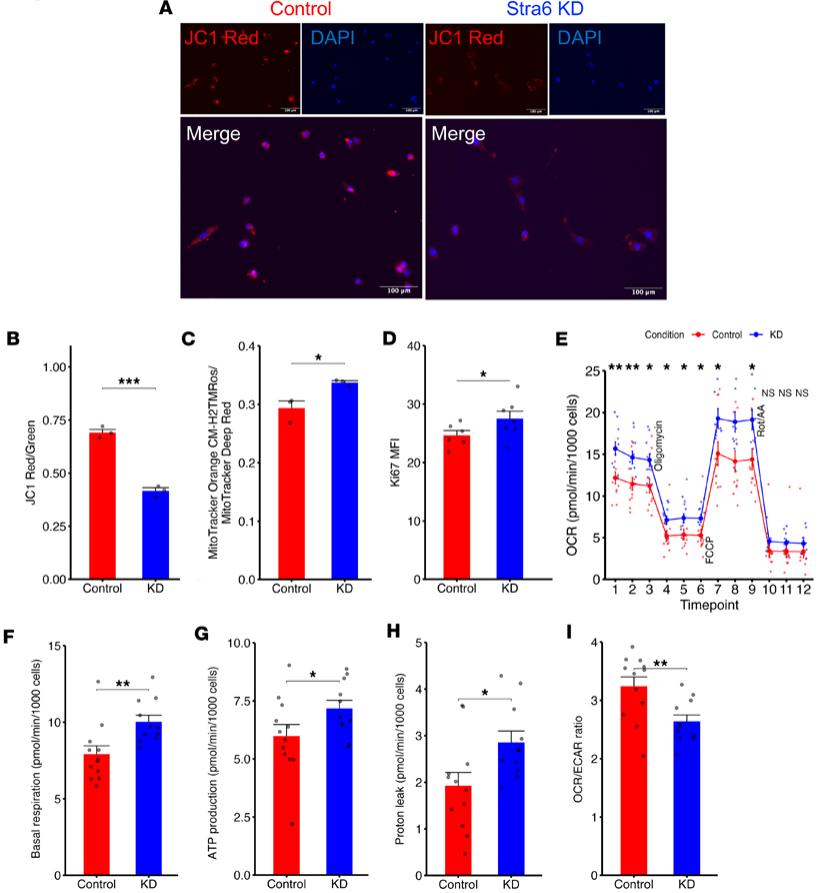
Stra6 loss induces mitochondrial dysfunction. (**A**) Representative image of mitochondrial membrane depolarization via JC-1 labeling (represented as ratio of red/green fluorescence) and representative images of red JC-1 aggregates indicating healthy, polarized mitochondria and Hoechst-counterstained nuclei (scale bars = 100 µm; Stra6-knockdown cells on the right and negative control siRNA cells on the left). (**B**) Quantification of JC-1 Red/Green ratio for knockdown and control, *n* = 3 wells per condition. Comparisons made via *t* test. Data are represented as averages across samples showing mean ± SEM. (**C**) Quantification of mitochondrial ROS using MitoTracker Orange CM-H2TMRos normalized to total mitochondrial stained by MitoTracker Deep Red (*n* = 3 wells per condition). (**D**) Quantification of Ki67 mean fluorescence intensity (*n* = 6 image fields across 2 wells per condition) after siRNA knockdown of Stra6 (blue) or negative control (red). (**E**) Line graphs of oxygen consumption rate (OCR) measured via Seahorse XFe96 Mito Stress Test in Stra6-knockdown cells (blue line, *n* = 11 wells) and negative control cells (red line, *n* = 12 wells) after injections of oligomycin, FCCP, and rotenone/antimycin A. (**F**–**I**) Quantification of OCR during basal cell respiration (**F**), change in OCR related to ATP production (**G**), proton leak (**H**), and OCR/ECAR ratio (**I**) in Stra6-knockdown cells and negative control cells. Comparisons of Seahorse Mito Stress parameters were made via *t* test. *P < 0.05, **P < 0.01, ***P < 0.001.

To further assess the influence of STRA6 on mitochondrial processes that assist with maintenance of quiescence, we knocked down *Stra6* using siRNAs in C2C12 myoblasts and confirmed knockdown levels with qPCR (Supplemental Figure 6A). To gain insights whether STRA6 loss disrupts mitochondrial dynamics, we utilized multiple live-cell mitochondrial dyes following *Stra6* knockdown including MitoTracker Orange CM-H_2_TMRos and JC-1, a mitochondrial membrane potential probe (51). We detected an increase in mitochondrial ROS (Figure 6C) and a decrease in mitochondrial membrane polarization (Figure 6, A–C) in *Stra6* knockdown cells compared with controls. We also observed that *Stra6* knockdown increased proliferation (Ki67; Figure 6D), as well as lipid peroxidation detected by BODIPY 581/591 C11, a lipid peroxidation sensor (Supplemental Figure 6, B and C) (52). Lastly, we found that knockdown of *Stra6* triggered an upregulation of p53, which is known to increase in scenarios of increased oxidative stress (Supplemental Figure 6D) (53).

To further determine how *Stra6* knockdown affected mitochondrial function, we utilized the Seahorse XF Mito Stress Test assay. *Stra6* knockdown resulted in increased OCR, ECAR, basal respiration, and ATP production, likely due to the increased bioenergetic demands of their heightened proliferative state (Figure 6, E–I). Notably, we also observed increased proton leak in *Stra6-*knockdown cells (Figure 6H), which has been similarly observed in aged mitochondria (54–56). *Stra6*-knockdown cells also exhibited a lower OCR/ECAR ratio indicative of a shift toward glycolysis (Figure 6I), which has also been observed in proliferative and aged myoblasts (4, 57, 58).

## Discussion

The pathological degeneration of MuSCs in old age is intrinsically linked to defects in mitochondria. We detected old-aged MuSCs lose VA, a powerful, exogenous antioxidant that is metabolized into RA. To directly examine the functional effects of reduced VA-derived RA signaling on MuSCs, we administered a VA-free diet to young animals. We found a VA-deficient diet induced premature MuSC activation and increases in redox-dependent metabolism and mitochondrial ROS. VA-free diet also was characterized by a loss of fatty acid oxidation and OXPHOS, resulting in loss of ability to maintain quiescence. Uniquely, the deleterious changes in MuSC mitochondria from VA-free diet did not associate with alterations in myofiber diameter, overall depletion of MuSCs or muscle regeneration, which is consistent with previous work that showed mitochondrial dysfunction in MuSCs do not manifest in regenerative defects in youth but are exacerbated in age (>24 months) (59, 60). Combining these results shows that loss of VA leads to stress responses that increase ROS and premature activation in MuSCs that, in turn, drive loss of ability to maintain quiescence.

Changes in vitamins and associated metabolites have significant influence on stem cell function, and epidemiological studies have shown that regular diet supplemented with higher intake of antioxidant vitamins renders improvements in health during aging (13, 19). However, connecting the functionality of adult stem cells with vitamin-derived pathways in aging remains underexplored. VA metabolites have been shown to interact with the α-subunit of mitochondrial ATP synthase and PPARs, which heterodimerize with RXRs to activate the oxidoreductase acyl-CoA oxidase (61–63). Activation of mitochondrial regulators of oxidative metabolism from VA would be consistent with our data, whereby administration of RARγ/RXRα agonists and ATRA resulted in increased expression of *PPAR*γ and *Ndufa2*, reduced proton leak and increased ATP production through OXPHOS. Moreover, treatment of aged MuSCs ex vivo with RARγ/RXRα agonists and ATRA reduced mitochondrial ROS and oxidative DNA damage and promoted fatty acid oxidation and reduced total mitochondrial density in vivo. We speculate that VA RA activation of PPARγ may promote fatty acid oxidation through or in synergy with Ret signaling, which has been shown to contribute to MuSC ability to maintain quiescence (64–68). Integrating these results show that administration of VA derived RA promotes oxidative metabolism in MuSCs, and restoration of this antioxidant augments mitochondrial function lost in old age.

VA is an indispensable, diet-derived nutrient that is metabolized into retinol, distributed in the bloodstream, and internalized by Stra6. We detected that *Stra6* expression was strongest in quiescent MuSCs and reduced with MuSC activation, differentiation, and in old age. Reductions in Stra6 coincided with loss of expression of multiple mitochondrial genes and likely affects how RAR/RXR factors interact with MyoD and MyoG (69–71). Similarly, knockdown of *Stra6* increased mitochondrial ROS and fragmentation and decreased mitochondrial depolarization and ATP production through OXPHOS. Given that Stra6 has been shown to be sensitive to Ca^2+^, these results suggest that retinol transport into and out of Stra6 may contribute to MuSC mitochondrial fission and fusion dynamics through a Ca^2+^-mediated mechanism (72, 73). However, future work is needed to determine changes in Ca^2+^ signaling with retinol levels and mitochondria.

### Limitations of study.

This work helps establish how VA derived RA and transport through Stra6 affects mitochondrial stress and function of MuSCs and clarifies the importance of this signaling axis in old age. Whether VA-derived RA affects aging of human MuSCs in a similar manner as our murine model remains to studied. Future work will address 3 key challenges. First, our vivo delivery of ATRA and CD3254/BMS961 could be improved by encapsulation in micro/nanoparticles, which have been shown to improve solubility, release kinetics, and improve cellular uptake (74). This would reduce the need to perform multiple injections and target MuSCs specifically. Next, our study focused on targeting a single RA receptor (RAR; RARγ) and retinoid receptor (RXRα) in old-aged MuSCs, but other RA and retinoid receptors may also influence MuSC behavior through interaction with muscle basic helix-loop-helix transcription factors. Since RA has been demonstrated to act as both an inducer of stemness and differentiation, future studies may evaluate the relative contributions of each RAR and RXR to Pax7 and MyoG-induced actions with high-throughput tools such as CRISPR/Cas9 (75, 76). Third, our work provides a foundation for targeting STRA6 and demonstrates that rescue of this receptor positively affects mitochondrial function. Since STRA6 also interacts with calmodulin, future work may evaluate the relationship between retinol, calcium, and mitochondrial membrane potential.

## Methods

Supplemental Methods are available online with this article.

### Sex as a biological variable.

Both male and female mice were used, and for experiments listed, mice were age- and sex-matched with littermate controls whenever possible.

### Animals and MuSC labeling.

Male and female Pax7^CreERT2^-Rosa26^nuclearTdTomato–nuclearGFP^, or Pax7-nTnG, mice were obtained from a breeding colony at the University of Michigan (UM). Male and female Pax7^CreERT2^-Rosa26^TdTomato^, or Pax7-TdTomato, were obtained from a breeding colony at UM. Male and female Pax7-MitoTAG were obtained from a breeding colony at the UM. Young and old-aged C57BL/6 WT mice were obtained from the Jackson Laboratory or from a breeding colony at UM. To activate Pax7-conditional reporter expression, mice were given 5 daily i.p. injections of 20 mg/mL tamoxifen in corn oil (75 mg/kg body weight) and allowed to recover 5 days before being euthanized for experiments. All mice were housed on a 12:12 hour light-dark cycle under UM veterinary staff supervision.

### Muscle digestion for MuSC isolations.

Mice were euthanized by CO_2_ asphyxiation followed by cervical dislocation as a secondary method to confirm death. The hindlimb muscles were dissected using sterile surgical tools, and muscles were kept separate by biological replicate. The dissected muscles were then minced into fine chunks (<1 mm^3^) with fine surgical scissors. The minced muscle was then added into 50 mL conical tubes containing 20 mL of digestion solution (2.5 U/mL dispase II and 0.2% mg/mL collagenase II in DMEM). The samples were incubated at 37°C on a shaker for 30 minutes before being mechanically dissociated by pipetting up and down several times with an FBS-coated 10 mL serological pipette and allowed to incubate at 37°C for an additional 30 minutes. The digestion enzymes were then quenched with 20 mL of stop solution per tube (20% heat-inactivated FBS in Ham’s F10 nutrient mix). The samples were then passed through a 70 μm cell strainer and centrifuged at 350*g* for 5 minutes at 4°C. The pellets were then washed and further processed according to the isolation procedures described below.

### FACS isolation of Pax7^CreERT2^-Rosa26^nuclearTdTomato–nuclearGFP^ MuSCs.

After muscle tissue digestion, the supernatant was aspirated and cell pellets were washed in 3–4 mL of FACS buffer (2% heat-inactivated FBS in HBSS), after which they were centrifuged once more at 350*g* and resuspended in an appropriate volume of FACS buffer for sorting (approximately 1 mL per sample). Just before sorting, the samples were incubated with DAPI at a final concentration of 1 μg/mL (protected from light) to stain for viability, and cell suspensions were passed through a 35 μm cell strainer. Sorting was done on a Sony MA900 cell sorter, and DAPI^–^/GFP^+^ MuSCs were collected into cold FACS buffer for immediate processing.

### FACS isolation of WT MuSCs.

After muscle tissue digestion, the supernatant was aspirated, and cell pellets were washed in 3–4 mL of FACS buffer. After centrifugation at 350*g*, the washed cell pellets were resuspended in 400 μL of FACS buffer (split across 2 separate 5 mL FACS tube per biological replicate) containing a mixture of primary antibodies: APC-CD31 (BioLegend, 102410, 1:400), APC-CD45 (BioLegend, 103112, 1:400), APC-Sca1 (BioLegend, 108112, 1:400), APC-CD11b (BioLegend, 101212, 1:400), APC-TER119 (BioLegend, 116212, 1:400), PE-CD29 (BioLegend, 102208, 1:200), and Biotin-CXCR4 (BD Biosciences, 551968, 1:200). Tubes were incubated on ice for 30 minutes, protected from light. Samples were washed, centrifuged at 350*g*, and resuspended in 200 μL of FACS buffer per tube containing PECy7-Streptavidin (eBioscience, 25-4317-82, 1:100). Samples were incubated for 20 minutes on ice, protected from light. Prior to sorting, samples were washed, centrifuged at 350*g*, resuspended in 1 mL of FACS buffer per biological replicate, and passed through a 35 μm cell strainer. Propidium iodide solution (Thermo Fisher Scientific, P3566, 1:1,000) was added to each sample tube just before sorting to stain for viability. Sorting was done on a Sony MA900 cell sorter, and PI^–^/APC^–^/PE^+^/PECy7^+^ MuSCs were collected into cold FACS buffer for immediate processing.

### MACS isolation of MuSCs.

After digestion of muscle tissue as described above, cell pellets were treated with Miltenyi Red Blood Cell Lysis Solution as described in the Miltenyi MACS Satellite Cell Kit isolation protocol. After RBC lysis and centrifugation at 350*g*, cells were washed with MACS buffer (PBS, 0.5% bovine serum albumin, 2 mM EDTA). Incubation with satellite cell kit microbeads and LS column separation was performed as described by the manufacturer. Afterward, cells were counted and further purified using the Miltenyi Anti-Integrin Alpha 7 microbeads according to the manufacturer’s instructions. Finally, cells were incubated in uncoated tissue culture flasks for 1 hour at 37°C and 5% CO_2_ as a final preplating step to remove remaining contaminating cell types. The supernatants from the flasks were collected and centrifuged at 350*g*, and cells were counted via hemocytometer and resuspended in appropriate volumes of media or buffer for further processing.

### Myoblast growth medium preparation.

MuSCs were cultured in myoblast growth medium consisting of Ham’s F10 nutrient mix (Thermo Fisher Scientific, 11550043), 1× Penicillin-Streptomycin (Thermo Fisher Scientific, 15140122), 20% heat-inactivated FBS (Thermo Fisher Scientific, 16140071), and 0.02 μg/mL of bFGF (Thermo Fisher Scientific, PHG0263).

### VA depletion.

To investigate the effect of VA depletion on muscle regeneration at the short-term time point, young (9 months and younger) Pax7-nTnG mice were fed a custom VA-deficient (VA-free) diet from Envigo Teklad (catalog TD.86143) ad libitum for a period of 8 weeks while control mice continued to receive normal mouse chow. For the long-term time point, young (3–5 months) Pax7-TdTomato mice were fed the same (VA-free) diet from Envigo Teklad (*n* = 5) as described above, while control animals (*n* = 3) received normal mouse chow. The VA-free chow was replenished every 1–2 weeks. In the final week, mice received daily tamoxifen injections as described above to label MuSCs and their progenitors after injury.

### Single-cell clonogenicity assay.

Seven- to 9-month-old Pax7-nTnG mice (2 males and 2 females per diet) were either fed VA-free or control chow as described above. Using the Sony MA900’s single-cell sorting mode and well-plate collection adapter, GFP^+^ MuSCs from the gastrocnemius and quadricep muscles from were sorted directly into 0.5% gelatin-coated 96-well plates containing 200 μL of myoblast growth media. Single-cell wells were kept in culture at 37°C and 5% CO_2_ for 5 days, and media were replaced every other day. Afterward, the wells were fixed with 4% PFA for 10 minutes at room temperature, washed with PBS, and labeled with DAPI (1 μg/mL in PBS) for 10 minutes at room temperature. Wells were imaged using Zeiss Axio Vert.A1 inverted microscope with a Colibri 7 LED light source and an AxioCam MRm camera and myogenic colonies were counted in Fiji.

### MitoTracker Deep Red, Ki67, and MyoD labeling of VA-free versus CTRL MuSCs.

Seven- to 9-month-old Pax7-nTnG mice (2 males and 2 females per diet) were either fed VA-free or control chow as described above. Ninety-six–well plates were coated with CellTak (Corning, 354240) with 50 μL of a 1:70 dilution in PBS and allowed to incubate for 20 minutes at room temperature before aspirating and seeding cells. In total, 2,000 nGFP^+^ MuSCs (isolated from the gastrocnemius and quadricep muscles) were seeded per well. Cells were incubated in myoblast growth medium at 37°C and 5% CO_2_ for 1 hour to allow the cells to settle and adhere to the CellTak. A total of 500 nM of MitoTracker Deep Red FM (Invitrogen, M22426) was prepared in prewarmed myoblast media. Half of the wells were incubated with MitoTracker for 30 minutes at 37°C and 5% CO_2_ and then washed 3 times with PBS before the entire plate was fixed with 4% PFA (10 minutes at room temperature). Permeabilization was performed by incubating with 0.1% Triton X-100 in PBS for 15 minutes at room temperature, followed by 3 washes in PBST (0.1% Tween-20 in PBS). Then, the cells were blocked with 1% BSA, 1% goat serum, and 22.52 mg/mL of glycine in PBST for 1 hour at room temperature. After 3 washes with PBST, the MitoTracker-labeled cells were incubated with PE-conjugated anti-Ki67 (Santa Cruz Biotechnology Inc., sc-23900, 1:50), and the remaining wells were incubated with Alexa Fluor 647–conjugated anti-MyoD antibody (Santa Cruz Biotechnology Inc., sc-377460, 1:50) in 1% BSA in PBST overnight at 4°C. Following overnight incubation with antibodies, cells were washed 3 times with PBS. Nuclei were counterstained with DAPI (1 μg/mL) for 10 minutes at room temperature. Cells were washed in PBS a final 3 times and left covered in 100 μL of PBS during imaging. MyoD-labeled wells were also imaged for Pax7-nGFP fluorescence. All the chemicals and serums used for immunofluorescence were sourced from Sigma-Aldrich. The 20× magnification images were acquired on a Zeiss Axio Vert.A1 inverted microscope with a Colibri 7 LED light source and an AxioCam MRm camera. Images were subsequently analyzed in Fiji.

### 8-OHdG labeling of VA-free versus CTRL MuSCs.

Three-month-old Pax7-nTnG mice (2 females per diet) were either fed VA-free or control chow as described above. Ninety-six–well plates were coated with CellTak (Corning, 354240) with 50 μL of a 1:70 dilution in PBS and allowed to incubate for 20 minutes at room temperature before aspirating and seeding cells. In total, 4,000 nGFP^+^ MuSCs (isolated from the gastrocnemius and quadricep muscles) were seeded per well. The plate was spun down at 50 RCF for 1 minute and then incubated in myoblast growth medium at 37°C and 5% CO_2_ for 30 minutes to allow the cells to settle and adhere to the CellTak. The plate was then fixed with 4% PFA (10 minutes at room temperature). Permeabilization and blocking was performed as described above. After 3 washes with PBST, the wells were incubated with rabbit IgG anti-8-OHdG (Bioss, bs-1278R) at a dilution of 1:100 in 1% BSA in PBST overnight at 4°C. Next, cells were washed 3 times with PBST and incubated with Alexa Fluor 647 goat anti–rabbit IgG (Invitrogen, a21245) in 1% BSA in PBST for 1 hour at room temperature. Following 3 PBS washes, nuclei were counterstained with DAPI (1 μg/mL) for 10 minutes at room temperature. Cells were washed in PBS a final 3 times and left covered in 100 μL of PBS during imaging. The 20× magnification images were acquired on a Zeiss Axio Vert.A1 inverted microscope with a Colibri 7 LED light source and an AxioCam MRm camera. Images were subsequently analyzed in Fiji.

### RNA-Seq library preparation for VA-free versus CTRL MuSCs.

Five- to 6-month-old Pax7-nTnG mice (all males) were either fed VA-free (*n* = 3) or control chow (*n* = 2) as described above. In total, 15,000–17,000 nGFP^+^ MuSCs per mouse (isolated from the gastrocnemius and quadricep muscles) were sorted on the Sony MA900 directly into 400 μL of Trizol for RNA lysis. After sorting the tubes were promptly vortexed to homogenize the lysates and then quickly frozen on dry ice before storing at –80°C until later processing. Trizol samples were thawed at room temperature, and the RNA-containing aqueous phase was separated by adding 0.2 mL of chloroform per 1 mL of Trizol to each tube, vortexing for 10 seconds, and incubating at room temperature for 2 minutes. The sample tubes were then centrifuged for 15 minutes at 350*g* at 4°C and the top aqueous phase was carefully transferred to a new tube, where it was mixed with 1.5 volumes of 100% ethanol. At this point, biological replicates were split into 2 technical replicates (A and B) to account for spin column volume. The RNA was purified using the Qiagen RNeasy Micro kit, starting at step 5 of the manufacturer’s protocol, “Purification of Total RNA from Animal and Human Cells.” RNA was eluted into 12 μL of RNase-free water. RNA concentration and quality was measured using the Agilent Bioanalyzer 2100 RNA 6000 Pico kit, following the manufacturer’s protocol. Samples with a RIN of 9 or higher and sufficient RNA concentrations were selected for downstream library prep (VA-free diet replicates 1B, 2A, 3A, and 3B and control diet replicates 1A, 1B, 2A, and 2B).

The Takara Bio SMART-Seq v4 Ultra Low Input RNA Kit for Sequencing was used for cDNA synthesis. Following the manufacturer protocols, 5 ng of input RNA was used per sample and 10 PCR cycles were run. Quality control and concentration measurements were performed using the Agilent Bioanalyzer 2100 High Sensitivity DNA kit. Illumina library prep was performed with the Nextera XT DNA Library Preparation Kit and Nextera XT Index Kit, using an input of 500 pg of full-length cDNA amplicons per sample and following the kit protocols. Illumina libraries were then submitted to UM’s Advanced Genomics Core for pooling and sequencing on the Illumina NextSeq using 75 cycles and 75 bp single-end reads.

### Bulk RNA-Seq analysis.

Fastq files were tested for quality using FastQC, after which they were aligned to the Mus musculus GRCm38 (Ensembl release 96) reference transcriptome with Kallisto (77). Abundance matrices were imported with the tximport package into R for differential gene expression analysis using the DESeq2 package (78). Differentially expressed genes (*P*_adj_ < 0.05) were selected and genes of interest were plotted using the pheatmap package and ggplot2. Differentially expressed genes (*P*_adj_ < 0.05 and log_2_[fold change] >1) in the VA-free samples were then analyzed for GO term enrichment using the goseq package (79). Over- and underrepresented GO terms (*P* < 0.05) were selected and GO terms of interest were plotted on a bubble plot using ggplot2. Reaction fluxes were estimated using genome-scale metabolic modeling using the RECON1 model as previously reported (32, 37).

### BaCl_2_ injury and IHC on VA-free versus CTRL muscle tissue cross sections.

Three- to 6-month-old female Pax7-nTnG and Pax7-TdTomato were fed either a VA-free (*n* = 3–5) or control (*n* = 3) for 8 weeks. After 8 weeks, mice were anesthetized with 2% isoflurane and the TA muscle was injured via a 40 μL intramuscular injection of 1.2% BaCl_2_ in sterile PBS, while the left muscle served as an uninjured contralateral control or served as a saline injection. The injuries were allowed to regenerate for 7 and 28 days, at which points, mice were euthanized and TAs were carefully dissected, embedded in OCT in a cylindrical mold, and flash-frozen by submerging in isopentane cooled in a larger container of liquid nitrogen until OCT was solidified. Frozen tissues were sectioned on a CryoStar NX70 Cryostat at –20°C to obtain 10 μm cross sections, which were adhered to positively charged glass slides and allowed to dry for 30 minutes before proceeding to staining.

For the uninjured controls and 7 days time point, slides were fixed in acetone at –20°C in a coplin jar for 10 minutes and air-dried for 10 minutes. Then, sections were outlined with Vector Labs ImmEdge Hydrophobic Barrier PAP pen. After outlines were dried, sections were rehydrated in PBS for 5 minutes after room temperature. PBS was then gently aspirated off and slides were blocked in 10% goat serum in PBS for 1 hour at room temperature in a sealed, hydrated chamber. Blocking solution was carefully blotted off, and primary antibodies were diluted in 10% goat serum in PBS. Slides stained for CD68^+^ macrophages were incubated with a 1:50 dilution of rat IgG anti-CD68 (Bio-Rad, MCA1957) and a 1:500 dilution of rabbit IgG anti-Laminin 1+2 (Abcam, ab7463). Slides stained for Ly6G^+^ neutrophils were incubated with a 1:50 dilution of rat IgG anti-Ly6G (BD Biosciences, 550291) and a 1:500 dilution of rabbit IgG anti-Laminin 1+2 (Abcam, ab7463). Slides were incubated with primary antibodies in a sealed, hydrated chamber overnight at 4°C. Slides were then washed with PBS 3 times for 5 minutes. Secondary antibodies (Alexa Fluor 647 goat anti–rat IgG, Alexa Fluor 488 goat anti–rabbit IgG) were diluted 1:500 and DAPI was diluted to 1 μg/mL in PBS. Slides were incubated with secondary antibodies and DAPI for 1 hour at room temperature in the dark in a sealed, hydrated chamber. Slides were then washed with PBS 3 times for 5 minutes, and coverslips were mounted with a drop of ProLong Diamond Antifade Mountant. Coverslip mounting medium was allowed to cure overnight at room temperature before imaging.

For the long-term time point, slides were fixed in acetone at –20°C in a coplin jar for 10 minutes and air-dried for 10 minutes. Sections were then outlined with the hydrophobic pen and rehydrated in PBS for 5 minutes. Samples were incubated with MOM blocking solution for 1 hour following manufacturer’s instructions. After this, sections were incubated with primary antibodies overnight at 4°C (mouse IgG Sarcoglycan, 1:200, Leica Biosystems catalog A-SARC-L-CE; Rat IgG CD68 1:50, Rockland catalog 600-401-R10; Rabbit IgG RFP 1:50, Rockland catalog 600-401-379). Samples were then rinsed 3 times for 5 minutes each time with PBS and incubated with secondary antibodies (Alexa Fluor 488 goat anti-mouse, 1:500, catalog A-11001; Alexa Fluor 555 goat anti-rabbit, 1:500, catalog A-21428; and Alexa Fluor 647 goat anti-rat, 1:500, catalog A-21247; all Invitrogen) for 2 hours at room temperature. Tissues were rinsed again 3 times for 5 minutes each time with PBS and incubated with DAPI for 10 minutes, rinsed 2 times for 5 minutes each time, and mounted with 100 μL of ProLong Diamond Antifade Mountant. Mounting medium was allowed to cure overnight at room temperature before imaging.

Stained sections were imaged in duplicate per biological replicate at 20× on a Nikon A1si inverted confocal microscope using ND Large Image acquisition and stitching. Image files were analyzed in Fiji to calculate fiber area, centrally nucleated fibers, CD68^+^ stained area, and Ly6G^+^ stained area. For the long-term time point, 3 tissue sections across the length of the muscle were imaged per biological replicate at 20× on a Zeiss Laser Scanning Microscopy 900 with Airyscan 2. Outline of fibers was obtained using a trained model in CellPose with the cyto3 model as a base (80). Labels were then converted to regions of interest in Fiji to obtain fiber CSA (81). Positive Tdtomato^+^ fibers were extracted using the mean fluorescence intensity values of the Tdtomato^+^ channel for each fiber. Centrally nucleated fibers and cell density of CD68^+^ cells and Tdtomato^+^ were manually counted per tissue section.

### Stra6 expression in freshly isolated, activated, and differentiated MuSCs.

MuSCs were isolated via FACS from C57BL/6 WT mice (4 months old, 2 males and 1 female) as described above and pooled together to ensure high enough yield. Cells were then split into 3 time points with *n* = 2 replicates per time point. For the quiescent time point, cells were immediately pelleted for 10 minutes at 350 RCF, lysed in 350 μL of Qiagen Buffer RLT containing 1% 2-mercaptoethanol, and stored at –80°C. The remaining cells were cultured in a 24-well plate in myoblast growth medium for 3 days. At 3 days, 2 of the wells were lysed directly in the plate with 350 μL of Qiagen Buffer RLT containing 1% 2-mercaptoethanol and stored at –80°C, while the remaining wells were switched to differentiation medium (DMEM, 2% horse serum, 1% penicillin/streptomycin). After 3 days of differentiation, the final wells were lysed as described above. RNA isolation was performed using the Qiagen RNeasy Micro kit, following the kit’s instructions. RNA concentrations and quality were measured using a NanoDrop Spectrophotometer and Qubit Fluorometer using the RNA High Sensitivity Assay kit. Invitrogen’s SuperScript III First-Strand Synthesis System was used for cDNA synthesis (using the provided oligo dT primers), according to the manufacturer’s instructions.

*Stra6* and *Gapdh* primer assays were purchased from IDT (*Stra6*, Mm.PT.58.13854804; *Gapdh*, Mm.PT.39a.1) and were reconstituted in the manufacturer’s recommended volume of PCR-grade water for a 10× stock concentration. Quantitative PCR (qPCR) master mixes were prepared for a 50 μL reaction volume containing 25 μL of Power SYBR Green Master Mix (Applied Biosystems), 5 μL of primers, and 0.5 ng of cDNA template diluted in PCR-grade water. qPCR was performed on the Applied Biosystems QuantStudio 3 Real-Time PCR System with 2 technical replicates per sample and a blank water negative control to check for primer dimers. The ΔΔCt method was then used to find relative gene expression of *Stra6* between the time points.

### Statistics.

For imaging, qPCR, and Seahorse measurements, data were imported into RStudio, and statistical tests were performed using the RStatix package. For comparisons between 2 groups, comparisons were made with 1-sided Student’s *t* tests assuming unequal variance. For comparisons between more than 2 groups, comparisons were made by 1-way ANOVA followed by post hoc pairwise *t* tests with Bonferroni correction. Data were plotted using the ggplot package and annotated with the results of corresponding *t* tests.

### Study approval.

All procedures were approved by the University Committee on the Use and Care of Animals at UM and the IACUC in Ann Arbor, Michigan, USA (protocol no. PRO000010663) and were in accordance with the NIH.

### Data availability.

Bulk RNA-Seq and scRNA-Seq datasets (raw files and processed matrices) are publicly available on GEO (GSE268616 and GSE268617). Values for all data points in graphs are reported in the Supporting Data Values file.

## Author contributions

PMF, PD, VF, LA, JACM, VTW, SDG, CP, KI, and JAL performed experiments. PMF, PD, BAY, and SDG, analyzed data. PMF and CAA designed the experiments and wrote the manuscript with additions from other authors.

## Supplementary Material

Supplemental data

Supporting data values

## Version 1 03/25/2025

In-Press Preview

## Version 2 05/08/2025

Electronic publication
