# Supplementary material for: Vitamin A retinoic acid contributes to muscle stem cell and mitochondrial function loss in old age
Source: JCI Insight. 2025 Mar 25;10(9):e183706. doi: 10.1172/jci.insight.183706 (PMC12128968; doi:10.1172/jci.insight.183706)
Supplement: Supplemental data [file jciinsight-10-183706-s200.pdf]

# Vitamin A-Retinoic Acid Contributes to Muscle Stem Cell and Mitochondrial Function Loss in Old Age

Paula M. Fraczek[1,2], Pamela Duran[1,2], Benjamin A. Yang[1,2], Valeria Ferre[1,2], Leanne Alawieh[1,2], Jesus A. Castor-Macias[1,2], Vivian T. Wong[1,2], Steven D. Guzman[1,2], Celeste Piotto[1,2], Klimentini Itsani[1,2], Jacqueline Larouche[1,2], Carlos A. Aguilar[1,2,3,*]

[1]Dept. of Biomedical Engineering, University of Michigan, Ann Arbor, MI 48109, USA. [2]Biointerfaces Institute, University of Michigan, Ann Arbor, MI 48109, USA. [3]Program in Cellular and Molecular Biology, University of Michigan, Ann Arbor, MI 48109, USA. *To whom correspondence should be addressed: Carlos A. Aguilar, NCRC - University of Michigan, 2800 Plymouth Rd., 10-A183, Ann Arbor, Michigan 48109, USA. Phone: 734-764-8557; Email: caguilar@umich.edu.

**Supplemental Figure Captions**

**Supplemental Figure 1: FACS Isolation & quality control metrics for differential gene expression datasets of muscle stem cells fed a control or vitamin A free diet.** (A) Representative FACS plots showing negative (TdTomato+) and positive (GFP+) gates to isolate muscle stem cells (MuSCs) from $Pax7^{CreERT2}$-$Rosa26^{nTnG}$ mice where numbers within gates indicate percentage of cells within gate. **(B)** Spearman correlations for replicate libraries from control and MuSCs isolated from vitamin-A (VA) free diet. **(C)** PCA plot of replicate libraries for VA-free diet samples. (blue) and control diet samples (pink). **(D)** Heatmap of z-scores for top 60 differentially expressed genes related to mitochondrial GO terms with padj < 0.05 in VA-free and Control MuSCs. **(E)** Selected significant metabolic flux terms for both the VA-free and CTRL samples returned by metabolic flux model.

**Supplemental Figure 2: Disruption of microenvironment in VA-depleted skeletal muscle after barium chloride injury. (A)** Representative images of tibialis anterior muscle cross sections stained for laminin (green), nuclei (blue), and CD68+ macrophages (yellow; left) or Ly6G+ neutrophils (yellow; right) before (top) and after (bottom) barium chloride injury in young mice fed either a VA-free diet or control diet. **(B)** Fiber cross-sectional area distribution between mice fed control diet (red bars) and mice fed VA-free diet (blue bars) before (top) and after (bottom) barium chloride injury. Top inset shows the overall average fiber cross-sectional area in uninjured muscles, bottom inset shows percentage of centrally nucleated myofibers after barium chloride injury. **(C)** Quantification of $CD68^+$ macrophages in tibialis anterior cross sections before (top) and after (bottom) injury via barium chloride intramuscular injection. Cross sections were stained and imaged in duplicate for n=4 mice per diet for the uninjured condition, and n=3 mice per diet for the injured condition, and the $CD68^+$ area percentage of the technical replicates were averaged for each mouse before comparisons via t-test . **(D)** Quantification of $Ly6G^+$ neutrophils in tibialis anterior cross sections before (top) and after (bottom) barium chloride injury. Cross sections were stained and imaged in duplicate for n=4 mice for the uninjured condition, and n=3 mice for the injured condition and the $Ly6G^+$ area percentage of the technical replicates were averaged for each mouse before comparisons via t-test (p-value reported on plots). **(E)** Quantification of nGFP+ live cells (MuSCs) in uninjured muscle gated via FACS. Comparison made via t-test with n=4 mice per diet. Data are represented as averages across samples with error bars showing the S.E.M.

**Supplemental Figure 3: Vitamin A (VA)-depleted diet does not impact long-term muscle regeneration. (A)** Representative images of tibialis anterior muscle cross sections stained for sarcoglycan (green), nuclei (blue), CD68+ macrophages (pink) and Pax7-derived Tdtomato+ fibers 28 days after barium chloride injury in young mice fed either a control diet (top) or VA-free diet (bottom). **(B)** Fiber cross-sectional area distribution between mice fed control diet (red bars) and mice fed VA-free diet (blue bars). Inset shows the overall average fiber cross-sectional area. **(C)** Fiber cross-sectional area distribution of Tdtomato$^+$ fibers between mice fed control diet (red bars) and mice fed VA-free diet (blue bars). Inset shows the overall average fiber cross-sectional area. **(D)** Quantification of the percentage of TdTomato$^+$ fibers between control and treatment groups. **(E)** Quantification of the percentage of centrally nucleated fibers between control and VA-free diet. **(F)** Quantification of CD68$^+$ macrophages. **(G)** Quantification of Pax7$^+$-derived Tdtomato$^+$ muscle stem cells at the basal lamina of the fiber. Comparisons made via student's t-test with n=3 mice for control diet and n=5 for VA-free diet. Data are represented as mean $\pm$ S.E.M.

**Supplemental Figure 4: Rarɣ and Rxrα agonists in combination with ATRA enhances retinoic acid signaling, mitochondrial function & decreases cellular activation. (A-E)** Quantification of Stra6, Rarb, Rarg, Nduf2a, Pparg Log2(Fold Change Expression) found via RT-qPCR after treating C2C12 myoblasts with CD3254 and BMS961 agonists alone, ATRA alone, agonists with ATRA, or a DMSO vehicle control for 2 days. RT-qPCR was performed relative to Gapdh, with 3 sample wells per treatment condition and 2 technical replicates per sample. F**)** Line graphs of extracellular acidification rate (ECAR) measured via Seahorse XFe96 Mito Stress Test in C2C12s treated with ATRA and agonists (red line, n = 12 wells) and DMSO vehicle control (blue line, n=12 wells) after injections of oligomycin, FCCP, and Rotenone/Antimycin A. **(G-H)** Quantification of OCR during basal cell respiration, change in OCR related to ATP production, and coupling efficiency, respectively, in C2C12s treated with ATRA and agonists (red) and DMSO vehicle control (blue). Comparisons of Seahorse Mito Stress parameters were made via t-test. **(J-L)** Quantification of mitochondrial coupling efficiency, change in OCR related to ATP production, and OCR/ECAR ratio, respectively, in C2C12s treated with agonists alone, ATRA alone, agonists with ATRA, or a DMSO vehicle control for 2 days. Comparisons of Seahorse Mito Stress parameters were made via t-test (n=12 wells per condition). Data are represented as averages across samples with error bars showing the S.E.M (*: $p<0.05$, **: $p<0.01$, ***: $p<0.001$, ****: $p < 0.0001$). **(M)** Representative images of EdU labeling in MACS-isolated young MuSCs treated with DMSO vehicle control (top) or agonists and ATRA (bottom). EdU is shown in red and nuclear counterstain in DAPI is shown in blue. Scale bar = 20 µm. **(N-Q)** Quantification of Stra6, MyoD, MitoTracker Orange CM-H2TMRos, and 8-OHdG (respectively) mean fluorescence intensity for old aged (n=24 months) MuSCs treated with agonists alone, ATRA alone, agonists with ATRA, or a DMSO vehicle control for 3 days. Comparison made via t-test with n=4 wells per treatment. Comparisons were made via t-test.

**Supplemental Figure 5: Profiling response of old aged muscle stem cells after Retinoic Acid + ATRA treatment shows shifts muscle stem cell metabolism. (A)** Violin plots of quality control metrics of single cell RNA-Seq datasets, whereby nFeature (genes per cell), nCount (number of unique molecular identifiers - UMIs per cell), and percent.mt (percentage of mitochondrial gene transcripts per cell). **(B)** Dot plot of marker gene expression used to annotate cell clusters. **(C)** UMAP of all cell populations split by treatment. Control-treated muscle on the left,

Agonist+ATRA-treated muscle on the right. **(D)** Cell-type proportion tests determined from scProportionTest package plotted as log2 fold difference of cell proportions. Dots in pink are statistically significant with an FDR < 0.05. **(E)** Re-clustering of muscle stem cells shows 6 subclusters. **(F)** Heatmap of marker genes from each subcluster from I). **(G)** Bar plot of selected differentially enriched GO Term Biological Processes among downregulated genes in the Agonist+ATRA-treated MuSC cluster, found via EnrichR. **(H)** Scatter plot of differentially expressed reaction pathways across various metabolic subsystems found via Compass. Cohen's D test statistic is plotted on the x-axis. Opaque dots have a Wilcoxon-adjusted p-value < 0.1, translucent dots are not statistically significant.

**Supplemental Figure 6: Knockdown of Stra6 in C2C12 myoblasts activates P53 and lipid peroxidation. (A)** qPCR verification of knockdown of Stra6 using Lipofectamine RNAiMax delivery of 2 different siRNA sequences (IDT mm.Ri.Stra6.13.1 and mm.Ri.Stra6.13.2) into C2C12 cells. Upon quantifying the change in ΔΔCt and log2(expression fold change), Stra6 siRNA 2 (IDT mm.Ri.Stra6.13.2) was selected for further knockdown experiments. qPCR was run with 2 biological replicates and 2 technical replicates per condition. Statistical significance was found via t-tests comparing the log2(fold change) values for each siRNA to the empty lipofectamine vehicle control. **(B,C)** Quantification of lipid peroxidation via C11-Bodipy labeling (calculated as a ratio of red/green fluorescence; n = 3 wells per condition) and representative images showing unoxidized, membrane-localized lipids in red and Hoechst-counterstained nuclei (scale bars = 100 µm; Stra6 knockdown cells on the right and negative control siRNA cells on the left). **(D)** Quantification of P53 mean fluorescence intensity (n = 6 image fields across 2 wells per condition) after siRNA knockdown of Stra6 (blue) or negative control (red). Comparisons made via t-test. Data are represented as averages across samples with error bars showing the S.E.M (*: p<0.05, **: p<0.01, ***: p<0.001, ****: p < 0.0001).

## SUPPLEMENTAL INFORMATION FOR METHODS

### *Stra6 siRNA Knockdown Efficiency in C2C12 Myoblasts*

C2C12 myoblasts (ATCC CRL-1772) were seeded in a tissue culture-treated 24-well plate at a density of 1,000 cells/cm$^2$ in C2C12 growth medium (DMEM, 20% heat-inactivated FBS, without penicillin/streptomycin). Cells were allowed to attach for a few hours before introducing siRNAs. To test *Stra6* knockdown efficiency, cells were incubated with one of two different siRNA sequences from IDT (mm.Ri.Stra6.13.1 and mm.Ri.Stra6.13.2) or an empty lipofectamine control using Invitrogen's Lipofectamine RNAiMax Transfection Reagent (Cat. 13778075) and Gibco Opti-MEM I Reduced Serum Media (Cat. 31985070) according to the RNAiMax manufacturer's instructions. Transfection was performed in duplicate, and cells were incubated at 37°C and 5% $CO_2$ for 2 days. Cells were lysed directly in wells by pipetting up and down with 300 µL of Qiazol. RNA precipitation with chloroform and 100% ethanol was performed as described above and the aqueous phase was transferred and further purified using the Qiagen miRNeasy Micro Kit according to the manufacturer's instructions. RNA quality and concentration was measured using a NanoDrop spectrophotometer and Qubit Fluorometer using the RNA High Sensitivity Assay kit. cDNA was synthesized using Invitrogen's SuperScript III First-Strand Synthesis System (with provided oligo dT primers) according to the manufacturer's instructions. RT-qPCR using *Stra6* and *Gapdh* primers was performed as described above with 2 technical replicates per sample and gene and 4 ng of template cDNA per reaction and run on the Applied Biosystems QuantStudio 3

Real-Time PCR System. Stra6 Log2(Fold Change Expression) was calculated using the ΔΔCt method and IDT sequence mm.Ri.Stra6.13.2 was selected for further Stra6 knockdown experiments due to its higher efficiency.

***Live-Cell Staining and Immunofluorescence Imaging From Stra6 Knockdown C2C12 Myoblasts***

JC-1 dye (Invitrogen Cat. M34152) for mitochondrial membrane potential was prepared by dissolving in DMSO to a stock concentration of 1 µg/µL. MitoTracker Orange CM-H2TMRos (Invitrogen Cat. M7511) and MitoTracker Deep Red FM (Invitrogen Cat. 22426) were prepared by dissolving in DMSO to a stock concentration of 1 mM. CellMask Green Actin dye was dissolved in DMSO to a stock concentration of 1mM. BODIPY 581/591 C11 Lipid Peroxidation Sensor (Invitrogen Cat. D3861) was prepared by dissolving in DMSO to a stock concentration of 10 mM.

12 mm-diameter glass coverslips for mitochondrial morphological imaging were prepared by washing with 70% ethanol and autoclaving, followed by carefully transferring into a 24-well plate with sterile forceps and incubating with 500 µL 0.1% bovine gelatin solution (Sigma Aldrich Cat. 9391 diluted in distilled water and autoclaved to dissolve) per well for 10 minutes at 37°C, aspirating, and air-drying before seeding cells.

C2C12s were seeded at a density of 1,000 cells/$cm^2$ either directly into a tissue culture-treated 24-well plate or onto glass coverslips coated in 0.1% gelatin within a 24-well plate. *Stra6* knockdown was performed as described above (using IDT mm.Ri.Stra6.13.2 and IDT NC-1 negative control siRNA). Cells were incubated for 72 hours after transfection.

Live cell staining was performed as follows: JC-1 staining was performed by incubating cells with a 1:1000 dilution of JC-1 stock dye and 1:1000 dilution of Hoechst solution (BD Biosciences Cat. 561908) in phenol-free, serum-free DMEM (Gibco Cat. 21063029) for 30 minutes at 37°C and 5% $CO_2$. Mitochondrial ROS staining was performed by incubating cells with 500 nM of MitoTracker Orange CM-H2TMRos, 500 nM of MitoTracker Deep Red FM, and a 1:1000 dilution of Hoechst in phenol-free, serum-free DMEM for 30 minutes at 37°C and 5% $CO_2$. BODIPY 581/591 C11 staining was performed by incubating with 10 µM of BODIPY and a 1:1000 dilution of Hoechst in phenol-free, serum-free DMEM for 30 minutes at 37°C and 5% $CO_2$. After incubation, stains were aspirated off and replaced with fresh phenol-free, serum-free DMEM and plates were returned to the incubator to equilibrate for 15 minutes. Cells were then imaged on an EVOS M7000 Imaging System with an onstage incubator at 37°C, 5% $CO_2$, and 80% humidity. Images were analyzed in Fiji to find the mean fluorescence intensity of each channel.

Cells grown on coverslips were stained with 500 nM of MitoTracker Deep Red FM, 1 µM of CellMask Green Actin dye, and a 1:1000 dilution of Hoechst in phenol-free, serum-free DMEM for 30 minutes at 37°C and 5% $CO_2$. Cells were then fixed in 4% PFA in PBS for 15 minutes at room temperature and washed 3 times in PBS for 5 minutes. After aspirating off excess PBS, coverslips were mounted onto glass slides with a drop of ProLong Diamond Antifade Mountant, which was allowed to cure overnight at room temperature. Coverslips were then imaged on a Nikon A1si inverted confocal microscope at 60x magnification to visualize mitochondria within single cells. Images were analyzed in Fiji using the 3DSuite plugin to segment and measure mitochondria.

Remaining cells grown in 24-well plates were fixed in 4% PFA for 15 minutes at room temperature, then permeabilized with 0.1% Triton X-100 in PBS for 15 minutes at room temperature. Triton X-100 was aspirated off and cells were washed 3 times in PBS. Then, cells

were blocked for 1 hour at room temperature with 1% BSA, 1% goat serum, and 22.52 mg/ml in PBST. Blocking solution was aspirated off and cells were washed 3 times with PBST. Cells were then incubated overnight at 4°C with a 1:100 dilution of Rabbit IgG anti-P53 (Proteintech Cat. 10442-1-AP) and 1:50 dilution of PE-Mouse IgG1 anti-Ki67 (Santa Cruz) in 1% BSA in PBST. Primary antibodies were aspirated off and washed 3 times with PBST. Cells were then incubated for 1 hour at room temperature with a 1:500 dilution of Alexa Fluor 488 Goat anti-rabbit IgG (Invitrogen Cat. A11008) in 1% BSA in PBST. The secondary antibody was washed off and cells were washed 3 times with PBS before incubating with 1 µg/ml of DAPI in PBS and washing a final 3 times in PBS. Cells were then imaged on an EVOS M7000 Imaging System and were analyzed in Fiji to find the mean fluorescence intensity of each channel.

***Seahorse Extracellular Flux Mito Stress Test Assay on Stra6 Knockdown C2C12 Myoblasts***

C2C12s were seeded in a Seahorse XFe96 Cell Culture Microplate at a density of 700 cells/well and allowed to attach for 1 hour before Stra6 knockdown was performed (using IDT mm.Ri.Stra6.13.2 and IDT NC-1 negative control siRNA) as described above using the Lipofectamine RNAiMax instructions for 96-well plate volumes. After 2 days of incubation at 37°C and 5% CO2, the Seahorse XF Cell Mito Stress Test (Agilent Cat. 103015-100) assay was performed according to the manufacturer's protocol. Assay medium was prepared using the Seahorse XF DMEM assay medium pack (Agilent Cat. 103680-100) with 1mM pyruvate, 2 mM glutamine, and 10 mM glucose. Cells were equilibrated with assay medium for 1 hour at 37°C in a non-$CO_2$ incubator before assembling with the hydrated sensor cartridge. Oligomycin, FCCP, and Rotenone/Antimycin A were prepared in assay medium and loaded into the injection ports of the hydrated sensor cartridge to achieve final assay working concentrations of 1 µM, 2 µM, and 0.5 µM, respectively. The assay was run on an Agilent Seahorse XFe96 Extracellular Flux Analyzer using the Mito Stress Test template on the Seahorse Wave software. After the assay, cells in each well were fixed with 4% PFA in PBS for 15 minutes at room temperature, washed 3 times in PBS, incubated with 1 µg/mL of DAPI in PBS for 10 minutes at room temperature, and imaged at 4x on an EVOS M7000 Imaging System to count total cells per well for normalization of oxygen consumption and extracellular acidification rates. The Wave software was used to normalize and analyze flux data and generate output parameters, which were then plotted in R with ggplot2.

***Stra6 Immunofluorescence Imaging on Young and Aged Muscle Stem Cells***

MuSCs were isolated via MACS as described above from young (n=2 C57BL/6 females, 3-4 months old, pooled) and aged (n=2 C57BL/6 females, 22 months old, pooled) mice. A 96-well plate was coated with CellTak as described above. Cells were seeded, adhered by spinning the plate down at 100 RCF for 1 minute, fixed in 4% PFA in PBS for 15 minutes at room temperature, and washed 3 times in PBS. Cells were permeabilized with 0.1% Triton X-100 in PBS for 15 minutes at room temperature and washed 3 times in PBST. Cells were then blocked with 1% BSA, 1% goat serum, and 22.52 mg/mL of glycine in PBST for 1 hour at room temperature and washed 3 times with PBST. Cells were incubated overnight at 4°C with a 1:100 dilution of Rabbit IgG anti-Stra6 (Proteintech Cat. 22001-1-AP) in 1% BSA in PBST. Primary antibody was then aspirated, and cells were washed 3 times with PBST. Cells were then incubated with Alexa Fluor 488 Goat anti-Rabbit IgG (Invitrogen Cat. A-11008) in 1% BSA in PBST for 1 hour at room temperature. Cells were washed 3 times with PBS and incubated with 1 µg/ml of DAPI in PBS for 10 minutes at room temperature before imaging on an EVOS M7000 Imaging System and analyzing mean fluorescence intensity of Stra6 signal in Fiji.

***Treatment of C2C12 Myoblasts with Rarɣ, Rxrα agonists and ATRA***

C2C12 myoblasts were seeded in a tissue culture-treated 24-well plate at a density of 1,000 cells/cm$^2$ in C2C12 growth medium (DMEM, 20% heat-inactivated FBS, 1x penicillin/streptomycin). 100 µM stocks of CD3254 (Rxrα agonist, Tocris Cat. 3302), BMS961 (Rarɣ agonist, Tocris Cat. 3410), and all-trans Retinoic Acid (ATRA; Fisher Scientific, Cat. AC207341000) were prepared in DMSO. After cells attached, media was replaced with fresh C2C12 growth medium containing 100 nM of CD324, BMS961, and ATRA or an equal DMSO vehicle control (0.3% DMSO v/v). Cells were incubated for at least 2 days with daily supplementation of fresh media containing ATRA and agonists.

For RT-qPCR of *Stra6* expression after 2 days of treatment, cells were lysed in 250 µL of Qiazol, RNA was isolated using the miRNeasy Micro kit, cDNA synthesis was performed using the SuperScript III First-Strand Synthesis System, and RT-qPCR was performed to measure *Stra6*, *Rarg*, *Rarb*, *Pparg*, and *Ndufa2* expression relative to *Gapdh* (Table 1).

For the Seahorse Extracellular Mito Stress Test assay, C2C12s were seeded in a Seahorse XFe96 Cell Culture Microplate at a density of 700 cells/well and treated with agonists and ATRA or DMSO as described above. After 2 days of treatment, cells were switched to assay medium, and the Mito Stress Test assay was performed as described above.

***Treatment of Young Muscle Stem Cells with Rarɣ, Rxr∝ agonists and ATRA***

Pax7$^{CreERT2}$-Rosa26$^{CAG\text{-}LSL\text{-}EGFP\text{-}3xHA\text{-}OMM}$ or Pax7-MitoTAG mice (n=4, 3-5 months old) received daily injections of tamoxifen for 5 days to label the outer mitochondria membrane of MuSCs with an enhanced green fluorescence protein (EGFP). Cells were then isolated via MACS as previously described.(82) A 96-well plate with a glass bottom (Cellvis Cat. P96-1.5H-N, was coated with poly-l-lysine (Sigma Cat. P4832, 100 µg/ml in distilled water) for 30 minutes, rinsed 3x2 min with water and allowed to air dry for 30 minutes. The glass was then incubated with type I rat tail collagen (Gibco Cat. A1048301, 50 µg/ml in 20 mM of acetic acid) for 1 hour and rinsed 3x2 min with PBS. MuSCs were seeded at a density of 5,000 cells/well in myoblast growth medium and allowed to attach overnight at 37°C and 5% $CO_2$. The following day, myoblast growth medium was supplemented with 100 nM each of CD324, BMS961, and ATRA or an equal DMSO vehicle control (0.3% DMSO v/v) and medium with supplements was replenished daily for a total of 3 days in supplemented medium. After this, cells were incubated for 24 hours with EdU (5-ethynyl-2'-deoxyuridine) to assess alterations in MuSC proliferation with and without treatment. After incubation, cells were fixed with 4% PFA in PBS. Labeling of EdU$^+$ cells was done following manufacturer's instructions. Signal of EGFP in the mitochondria of MuSCs was further amplified by incubating the cells with a rabbit IgG GFP antibody (Abcam Cat. Ab13970, 1:500) overnight at 4°C and secondary incubation with Alexa Fluor 488 goat-anti rabbit (1:500) for 2 hours at room temperature. MuSCs were then incubated with DAPI for 10 min.

MuSCs were imaged at 40X using a CSU-W1 SoRa Spinning-Disk Confocal Microscope to profile EdU labeling across wells (n=18-20 image fields per well, 6 wells per condition). Z-stacks of single MuSCs were obtained with a 100X objective with Super-resolution mode to visualize individual mitochondrias. Cell density of EdU$^+$ cells was manually quantified using Fiji. Three-dimensional measurements of individual mitochondria were done using the Mitochondria Analyzer plugin in Fiji (n=10 cells/well, 5-6 wells analyzed per condition) (83).

***Treatment of Aged Muscle Stem Cells with Rarɣ, Rxrα agonists and ATRA***

MuSCs were isolated from 2 aged female C57Bl/6 mice (24 months old) via MACS as described above. A 96-well plate was coated with type I rat tail collagen as described above. Cells were seeded at a density of 4,000 cells/well in myoblast growth medium and allowed to attach overnight. Starting the following day, cells were treated with the agonists and ATRA as described above.

Immunofluorescence labeling of Stra6, MyoD, and 8-OHdG was performed after fixation with 4% PFA in PBS as described above and imaging was done on an EVOS M7000. Mean fluorescence intensity was analyzed in Fiji.

Live-cell staining and imaging of MitoTracker Orange CM-H2TMRos and MitoTracker Deep Red FM after treatment was performed as described above.

### *In Vivo Treatment of Aged Muscle with Rarγ, Rxrα agonists and ATRA*

Intramuscular injections were prepared by solubilizing 130 μM each of ATRA, CD324, and BMS961in sterile PBS with 0.6% DMSO v/v. Vehicle controls were prepared by dissolving 0.6% DMSO v/v in sterile PBS. Aged female C57Bl/6 mice (22-24 months old) were anesthetized via isoflurane inhalation and administered injections in the tibialis anterior (10 μL), gastrocnemius (30 μL), and/or quadriceps (50 μL). Injections were repeated every other day for a total of 3 days of injections. After the final day of injections, mice were allowed to recover for 3 days before being euthanized for muscle harvesting and processing. Cell suspensions from digested muscle were prepared as above and were either submitted for single-cell RNA sequencing (below) or purified for MuSCs using MACS as above. Isolated MuSCs were seeded on CellTak-coated 96-well plates and stained for MitoTracker Orange CM-H2TMROS, MitoTracker DeepRed, and Stra6 as described above. Images were acquired on an EVOS m7000 imaging system and analyzed with FIJI.

### *Single Cell RNA Sequencing of Treated Aged Muscles*

Aged female C57Bl/6 mice (24 months old) were given injections of ATRA, CD324, and BMS961 cocktail or vehicle control into the tibialis anterior, gastrocnemius, and quadriceps in both hindlimbs (n=2 per treatment) as described above. Mice were then euthanized, and the quadriceps and gastrocnemius muscles were dissected and digested to prepare a single cell suspension as described above. Cells were incubated with 1:1000 Propidium Iodide solution prior to sorting on a Sony MA900 cell sorter. $PI^-$ live cells were collected and biological replicates were pooled for sequencing. Libraries were prepared using the 10x Genomics Chromium Single Cell 3' High-Throughput kit v3.1 and sequenced on an Illumina NovaSeq X using the 10B 300 cycle kit and a 2 x 150 bp run configuration, targeting 50,000 reads per cell. All libraries were processed and sequenced at the same time to reduce batch variation.

### *Single Cell RNA-Seq Data Processing*

Raw sequencing data was converted to demultiplexed fastq files using Illumina's BCL Convert conversion software (v4.0). Fastq files were aligned to the mm10 genome using CellRanger v7.2.0.

Filtered feature-barcode matrices were imported into R and further processed with Seurat v4.4.0 (84). Low-quality cells and contaminating red blood cells were filtered out of the data by selecting for cells with 300-5000 features per cell, fewer than 20% mitochondrial (genes starting with "mt-") reads, and fewer than 0.25% hemoglobin (genes starting with "Hbb-") reads. The sample datasets were then merged and normalized using SCTransform. PCA and UMAP dimensionality

reductions were performed on the merged object and clusters were plotted by sample group to check for batch effects between the two libraries.

The filtered, separate Seurat objects underwent doublet removal using the DoubletFinder v2.0.4 package (85). An estimated doublet rate of 7.5% and a pN value of 0.2 were used to predict and filter out doublets. The filtered singlets were saved to as new Seurat objects. After initial QC filtering and doublet removal steps, we recovered 6,953 cells from the control samples and 6,906 cells from the ATRA/BMS961/CD3254-treated samples.

Singlet Seurat objects were merged, normalized using SCTransform, and clustered using PCA and UMAP dimensionality reductions again. The FindAllMarkers() function was called to identify marker genes between the clusters for cell type annotation. Cell types were annotated manually based on previously published single cell gene expression data (86). Cluster proportion testing was performed using the scProportionTest package to calculate log2FoldDifference of cell populations between the treated and control samples (87).

To find differentially expressed genes within the MuSC cluster between the two treatments, the MuSC cluster was subset out and the RNA assay was used to run the FindMarkers() function using DESeq2 as the test method. Differentially expressed genes were analyzed further with the EnrichR package for gene set enrichment analysis (GSEA) using the GO, KEGG, Reactome, and WikiPathways databases (88).

***Single Cell Genome-Scale Metabolic Flux Analysis with Compass***

Compass was used to estimate fluxes in metabolic pathway reactions based on single-cell gene expression data and the RECON2 metabolic model(49). Briefly, the MuSC cluster was subset, and the RNA assay data slot was exported as a .mtx file, along with .tsv files of cell barcodes and gene names according to Compass documentation. The Compass algorithm was run using Python 3.8 and IBM CPLEX Optimization Studio v22.11. The resulting reaction penalties matrix was used for differential analysis of metabolic reaction fluxes and subsystems using the Jupyter Notebook provided in the Compass documentation.

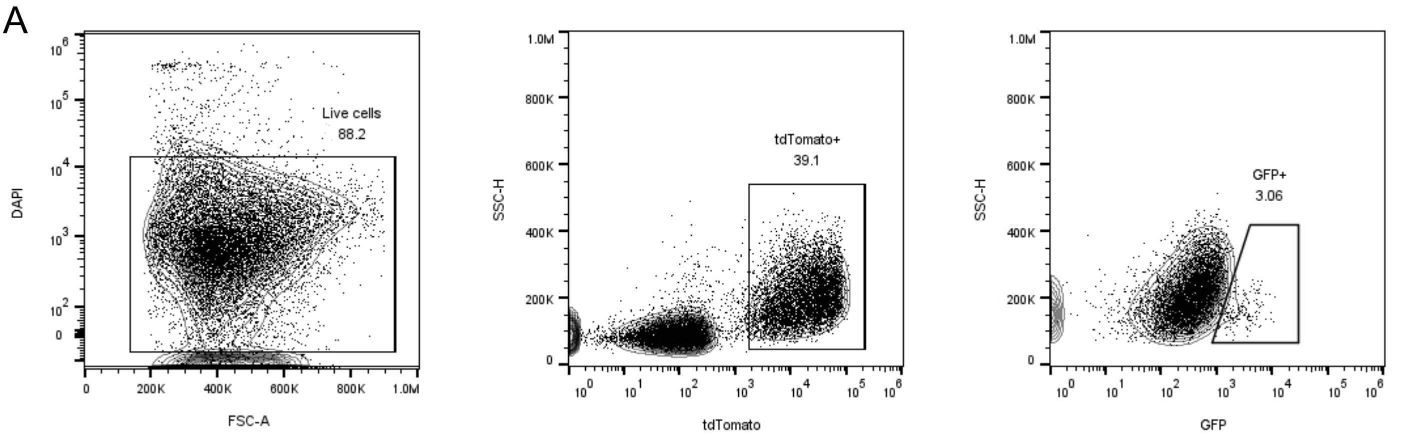


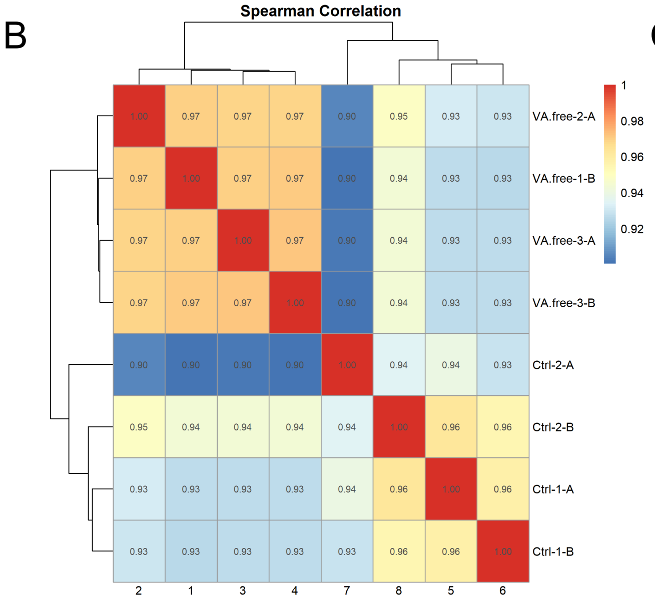


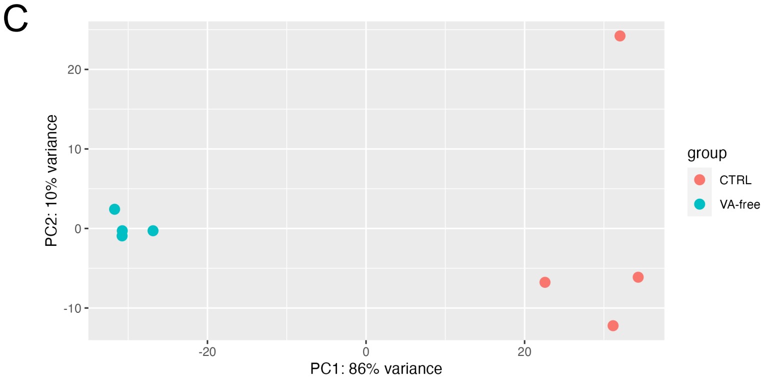


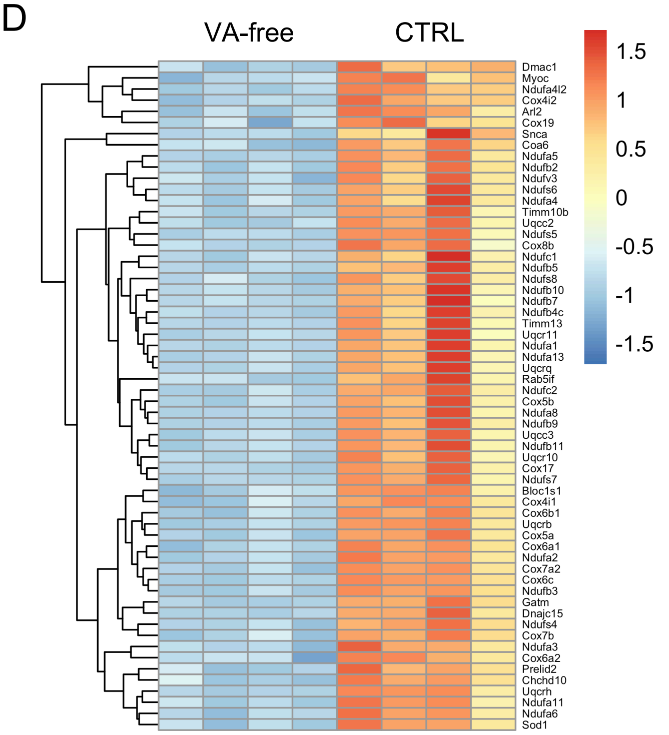


E

*Significant fluxes (z-score difference > 3)*

| | ID | Ctrl | VAFree | diff | diff_zscore | Name |
|---|---|---|---|---|---|---|
| Up in VA-Free | RDH3a | 6.51E-07 | 2.411861 | 2.41186 | 34.12515 | Retinol dehydrogenase (11-cis,NADPH) |
| | RADH3 | -2.14583 | -0.80263 | 1.343201 | 19.00545 | Retinal dehydrogenase |
| | RADH | -1.21464 | 6.51E-07 | 1.214636 | 17.18647 | Retinal dehydrogenase |
| | LDH_Lm | -0.57933 | -0.34677 | 0.232567 | 3.29187 | L-lactate dehydrogenase, mitochondria |
| | L_LACtm | -0.57933 | -0.34677 | 0.232567 | 3.29187 | L lactate transport mitochondrial |
| Up in Ctrl | PYRt2m | 3.368044 | 3.095132 | -0.27291 | -3.8598 | Pyruvate mitochondrial transport via proton symport |
| | RADH2 | 1.214636 | 6.51E-07 | -1.21464 | -17.1836 | Retinal dehydrogenase (NADPH) |
| | RADH4 | 2.145827 | 0.802626 | -1.3432 | -19.0026 | Retinal dehydrogenase (NADPH) |
| | RDH3 | 6.51E-07 | -2.41186 | -2.41186 | -34.1223 | Retinol dehydrogenase (11-cis,NADH) |

**Supplemental Figure 1**

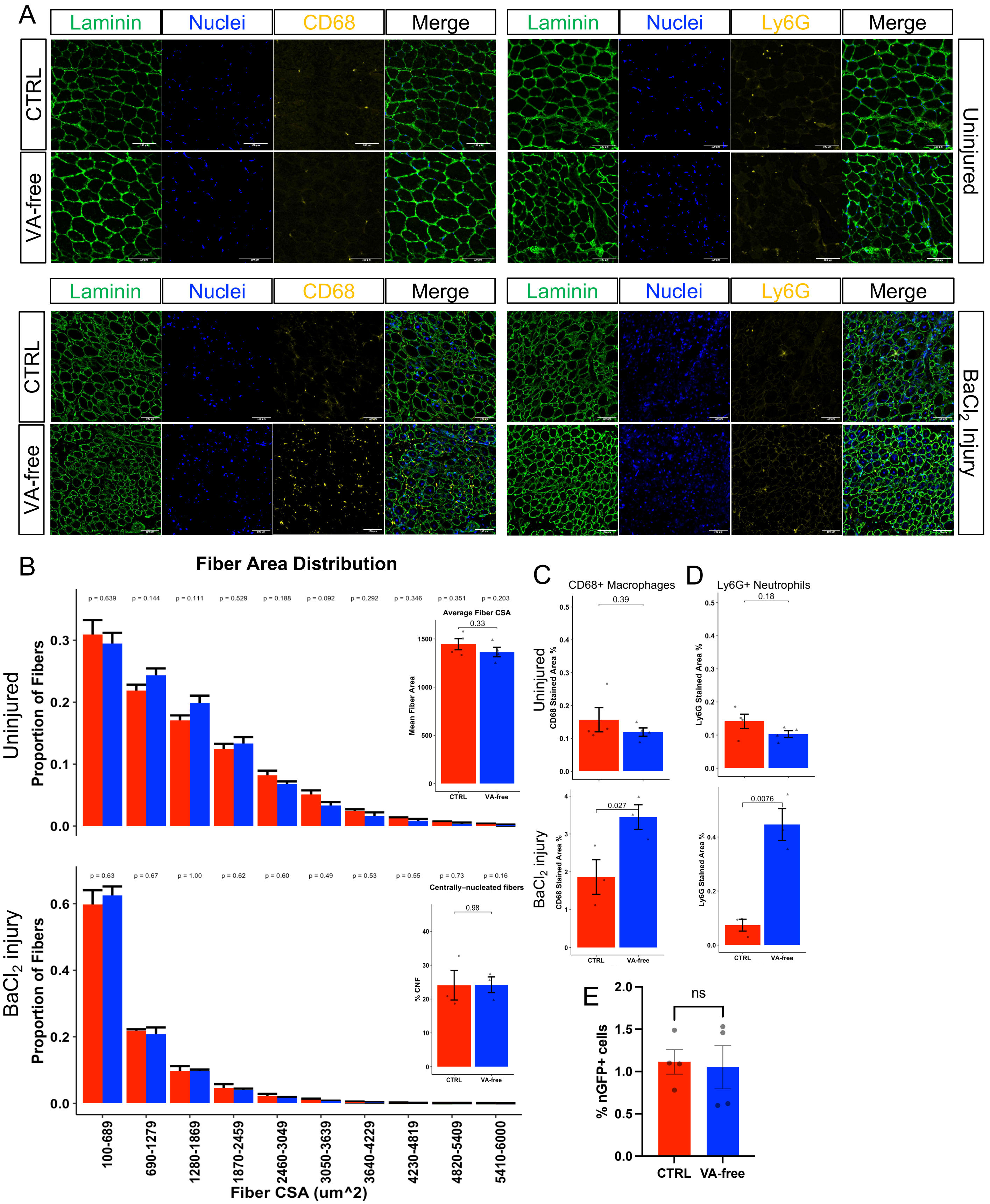


**Supplemental Figure 2**

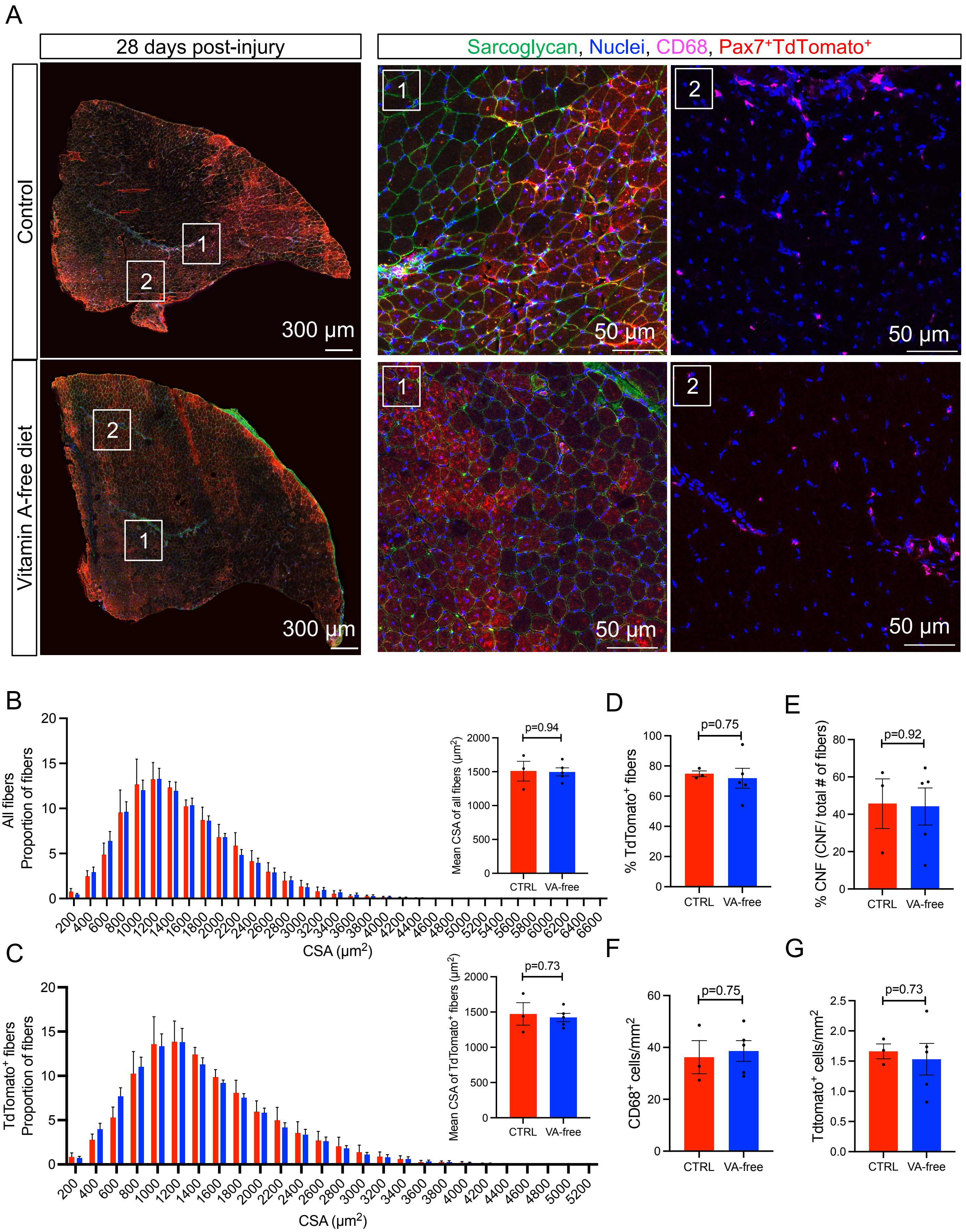

A
28 days post-injury
Sarcoglycan, Nuclei, CD68, Pax7+TdTomato+
Control
Vitamin A-free diet
1
2
300 μm
50 μm
B
All fibers Proportion of fibers
CSA (μm2)
Mean CSA of all fibers (μm2)
p=0.94
CTRL
VA-free
C
TdTomato+ fibers Proportion of fibers
Mean CSA of TdTomato+ fibers (μm2)
p=0.73
D
% TdTomato+ fibers
p=0.75
E
% CNF (CNF/ total # of fibers)
p=0.92
F
CD68+ cells/mm2
p=0.75
G
Tdtomato+ cells/mm2
p=0.73

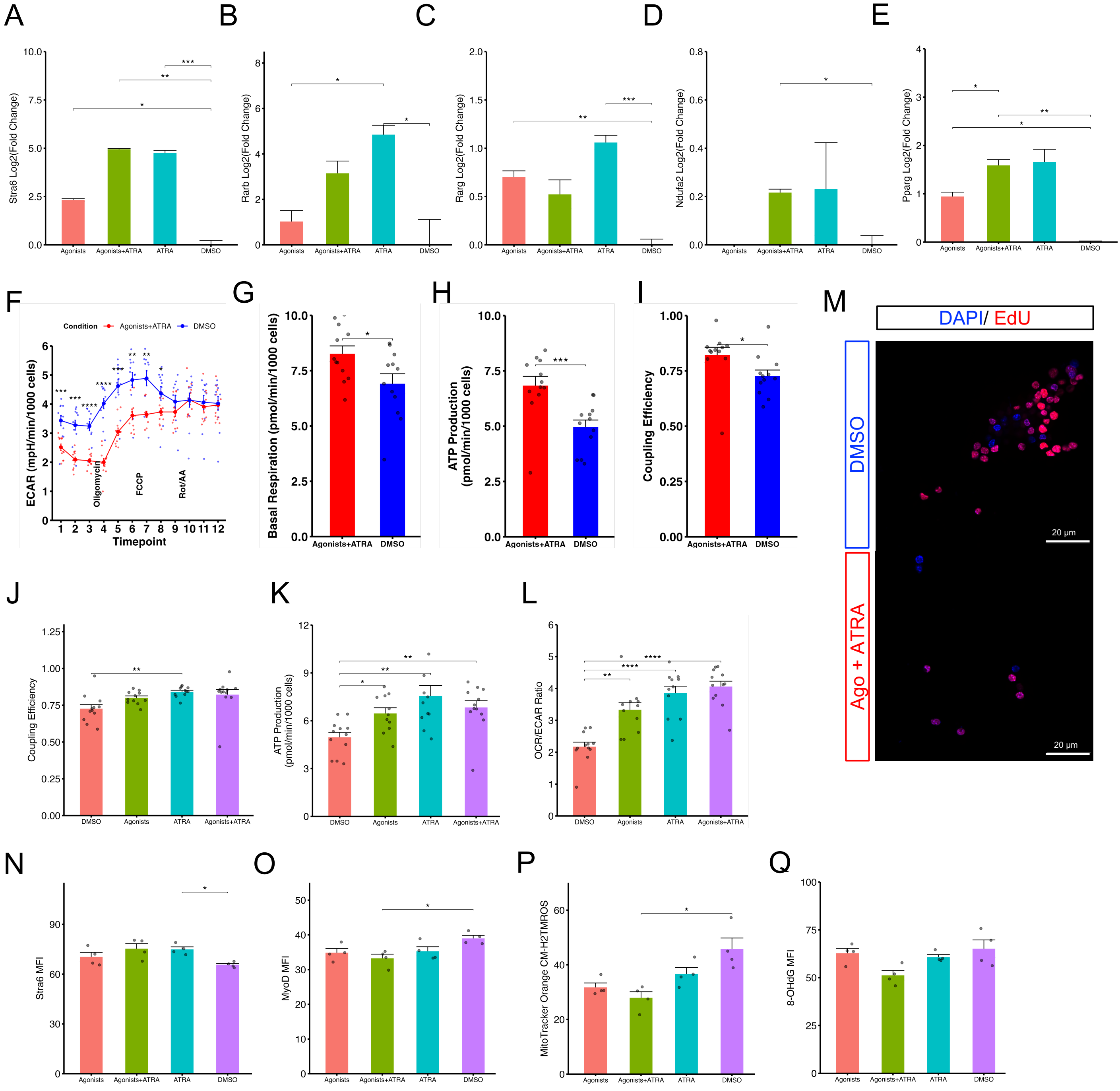


**Supplemental Figure 4**

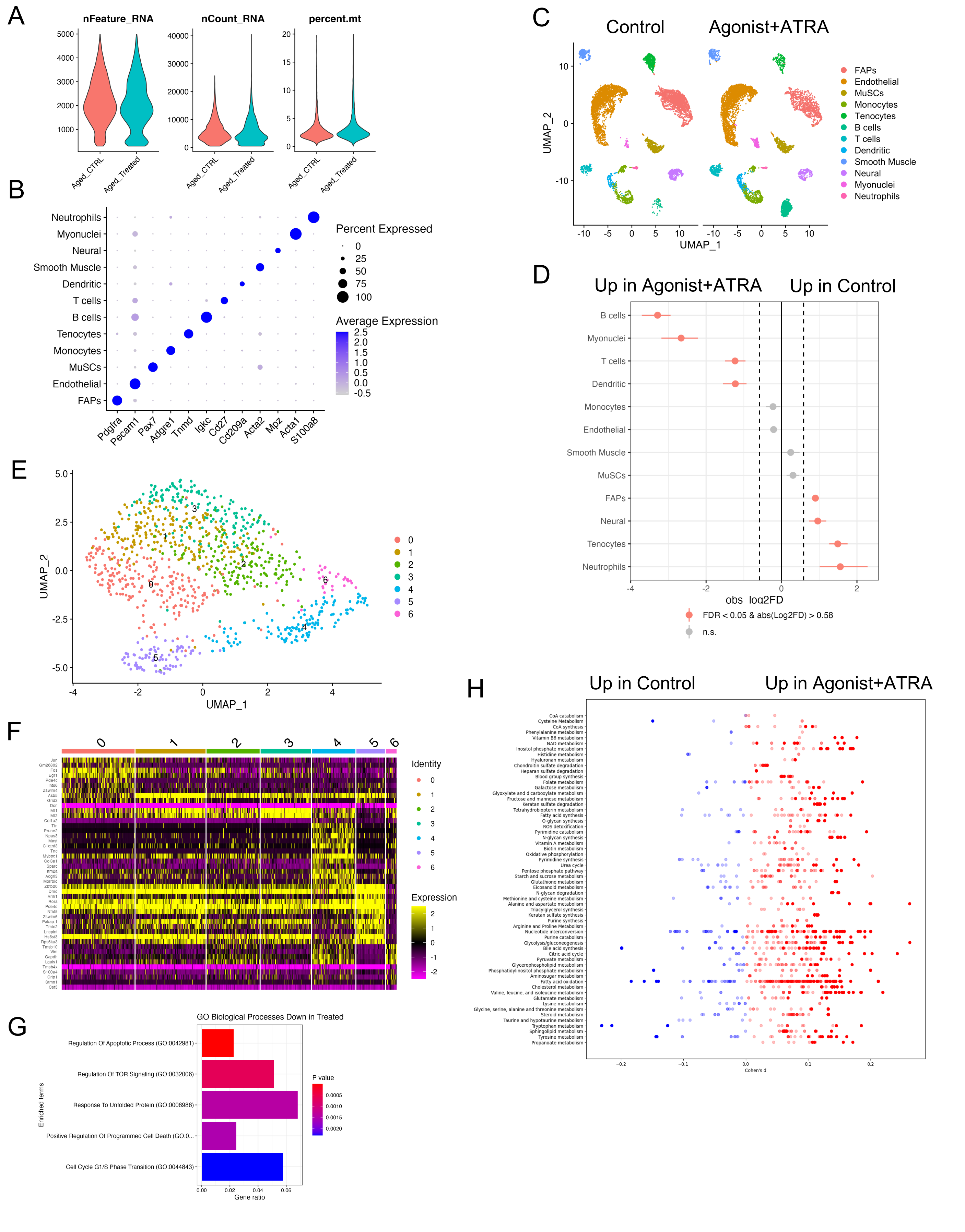


**Supplemental Figure 5**

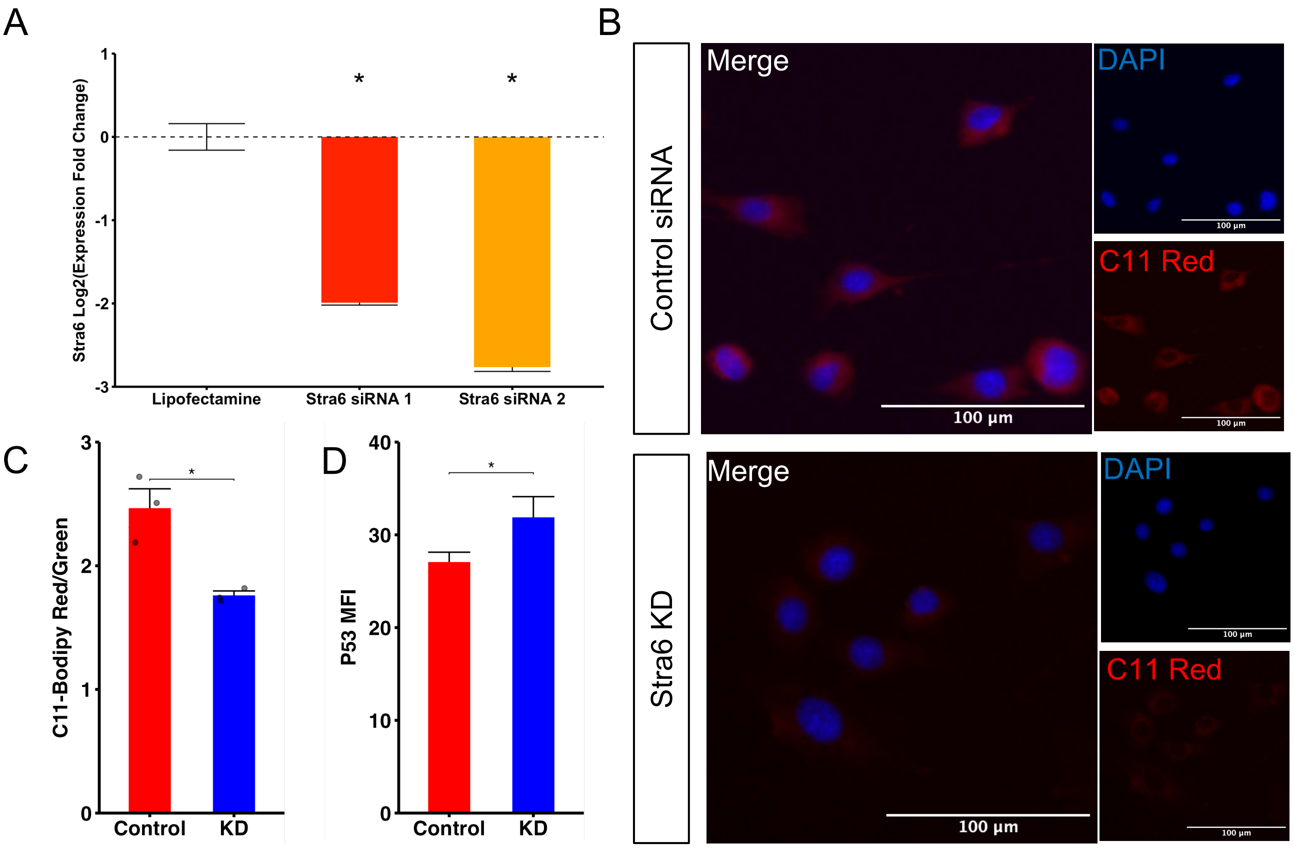


**Supplemental Figure 6**
